# Supplementary material for: Next-Gen GWAS: full 2D epistatic interaction maps retrieve part of missing heritability and improve phenotypic prediction
Source: Genome Biol. 2024 Mar 25;25:76. doi: 10.1186/s13059-024-03202-0 (PMC10962106; doi:10.1186/s13059-024-03202-0)
Supplement: Supplementary file 1 — Additional file 1. Additional text, figures and table. [file 13059_2024_3202_MOESM1_ESM.zip › Sup Figures 1 to 5.pdf]

Supplemental Figure 1.

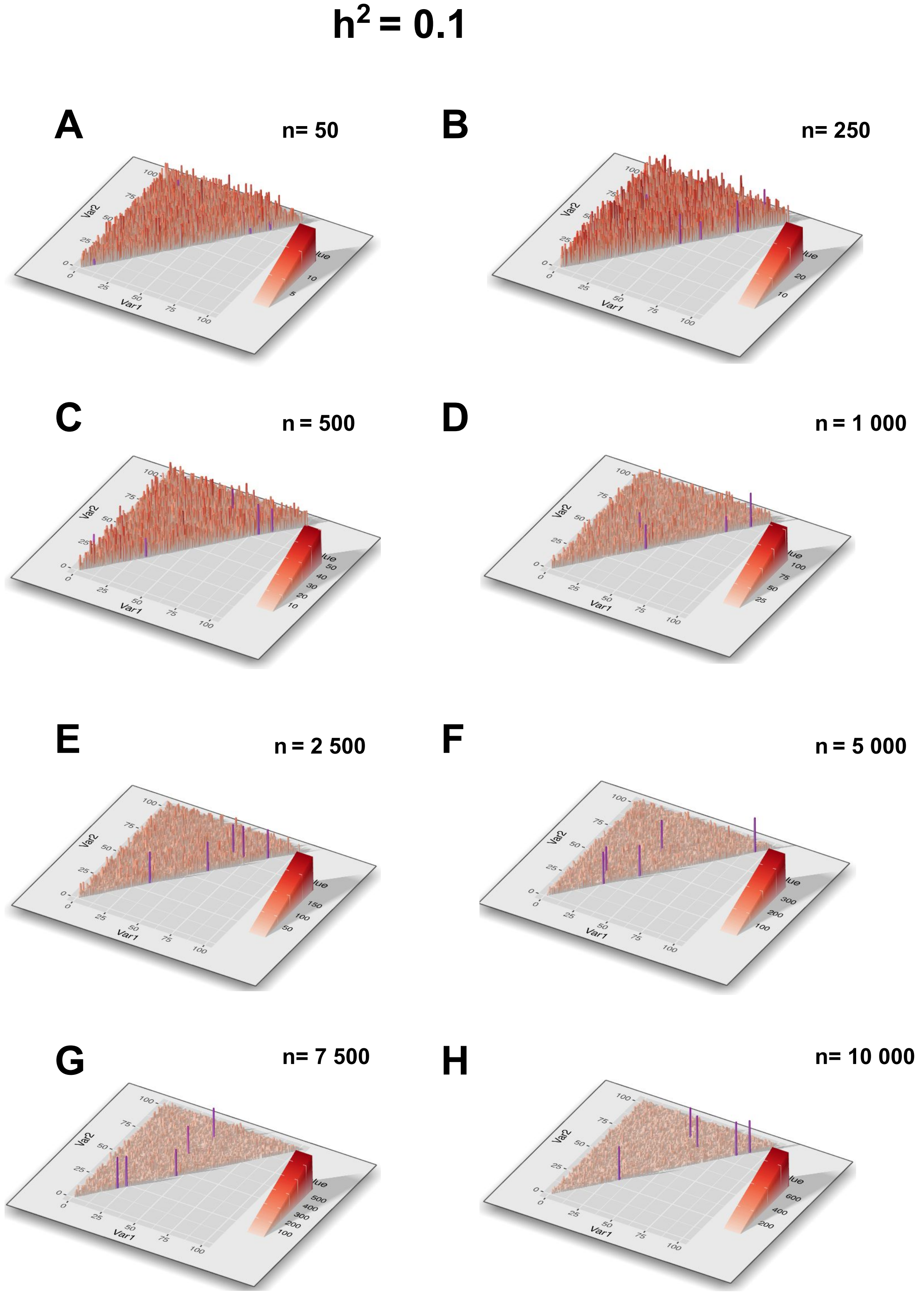

Supplemental Figure 1. Influence of the number of individuals on the power of epistatic signal detection.
Var1 (x axis) and Var2 (y axis) are a series of 100 SNPs. The triangle corresponds to SNPs combinations when the diagonal contains simple SNPs effects. z axis reports the estimated θ of simple SNPs (diagonal) and combinations (rest of the triangle). Genotype and phenotype data are simulated using specific and modulable parameters (see Github for details and code). Random noise has been added. Simulated signals are in purple. Simulations control heritability (h^2). The number of individuals (n) varies over the figures (Panels A-G, see details above panel). The number of individuals has a substantial effect on GWAS signal detection.

Supplemental Figure 1.

$h^2 = 0.2$

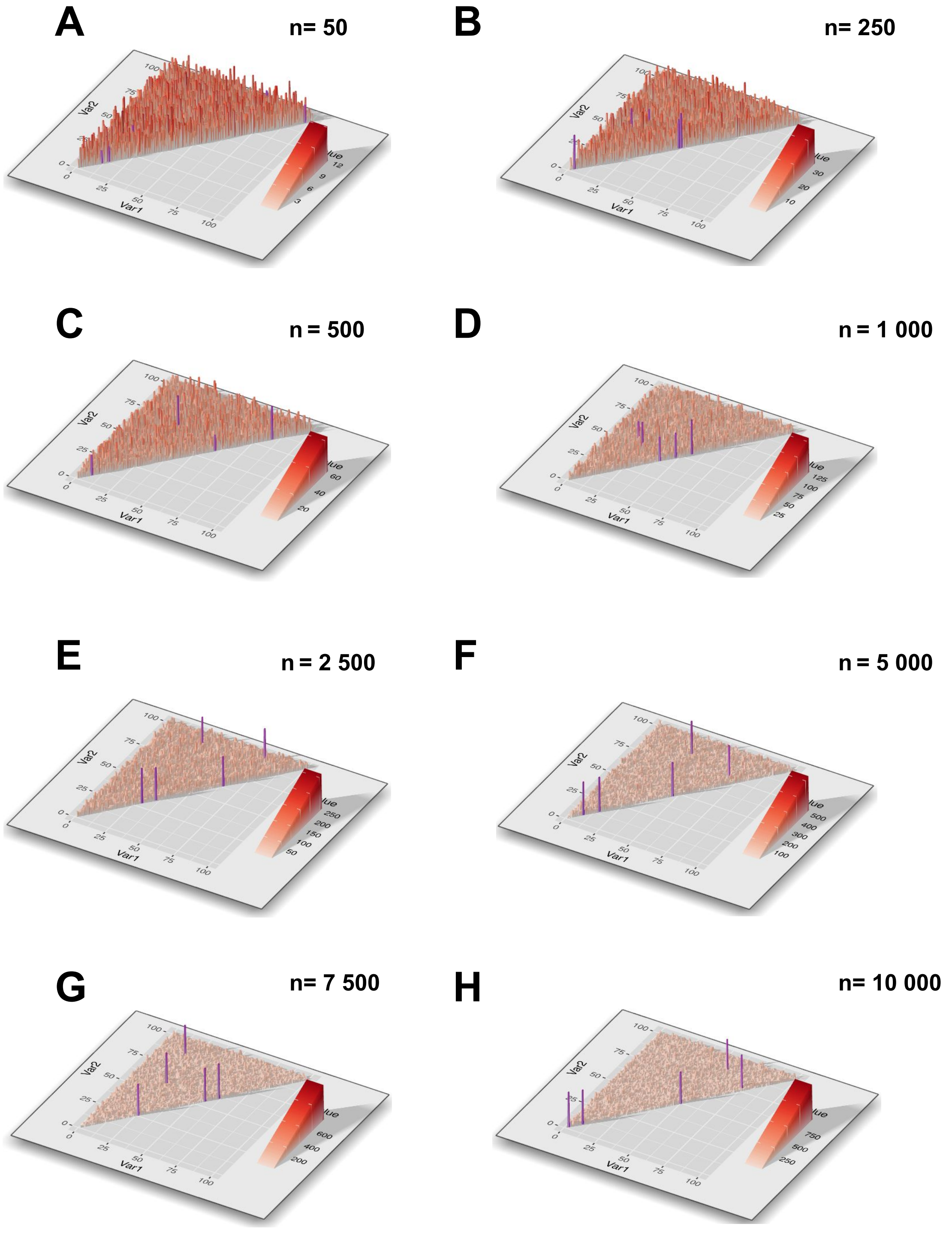

Supplemental Figure 1.

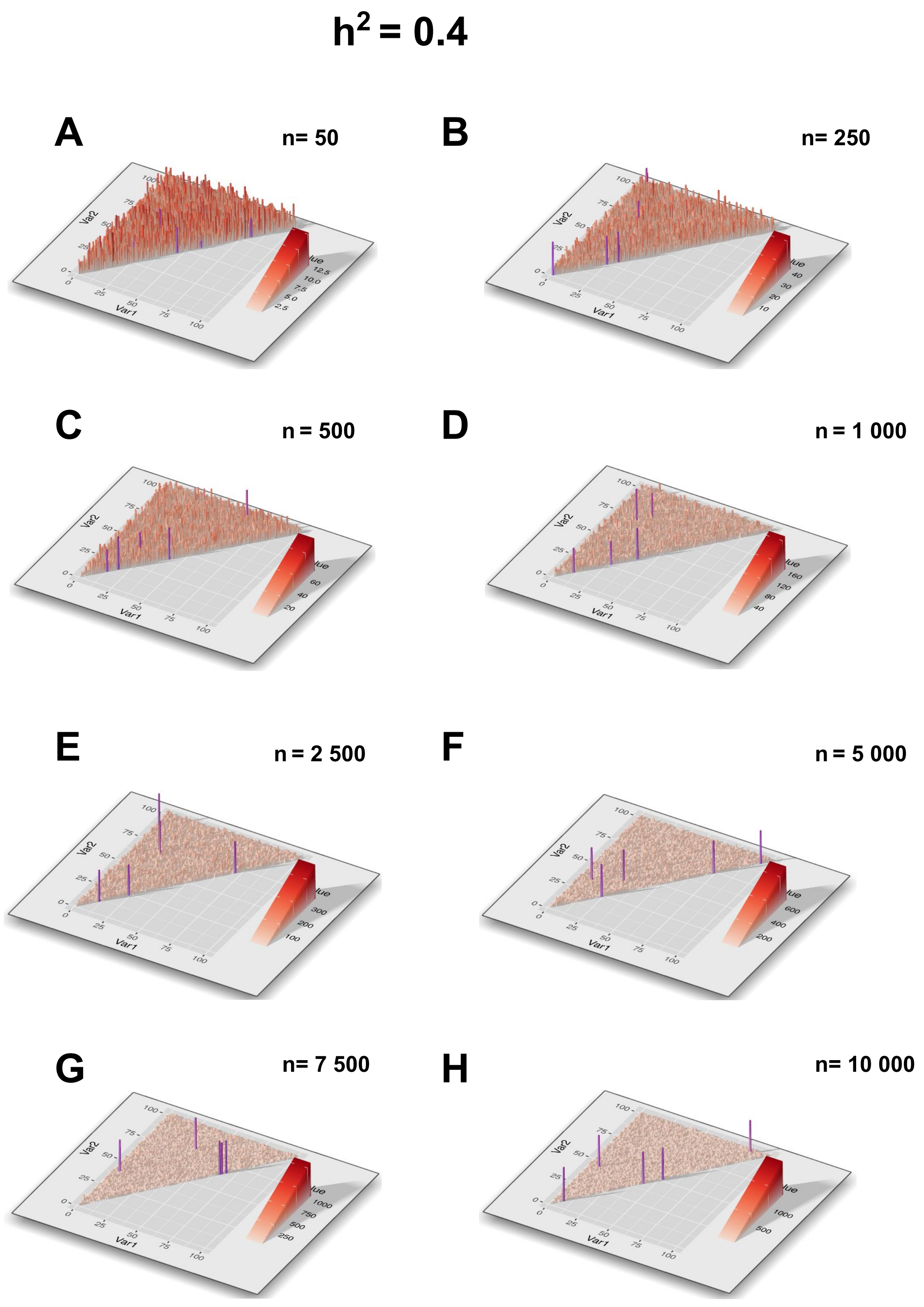

Supplemental Figure 1.

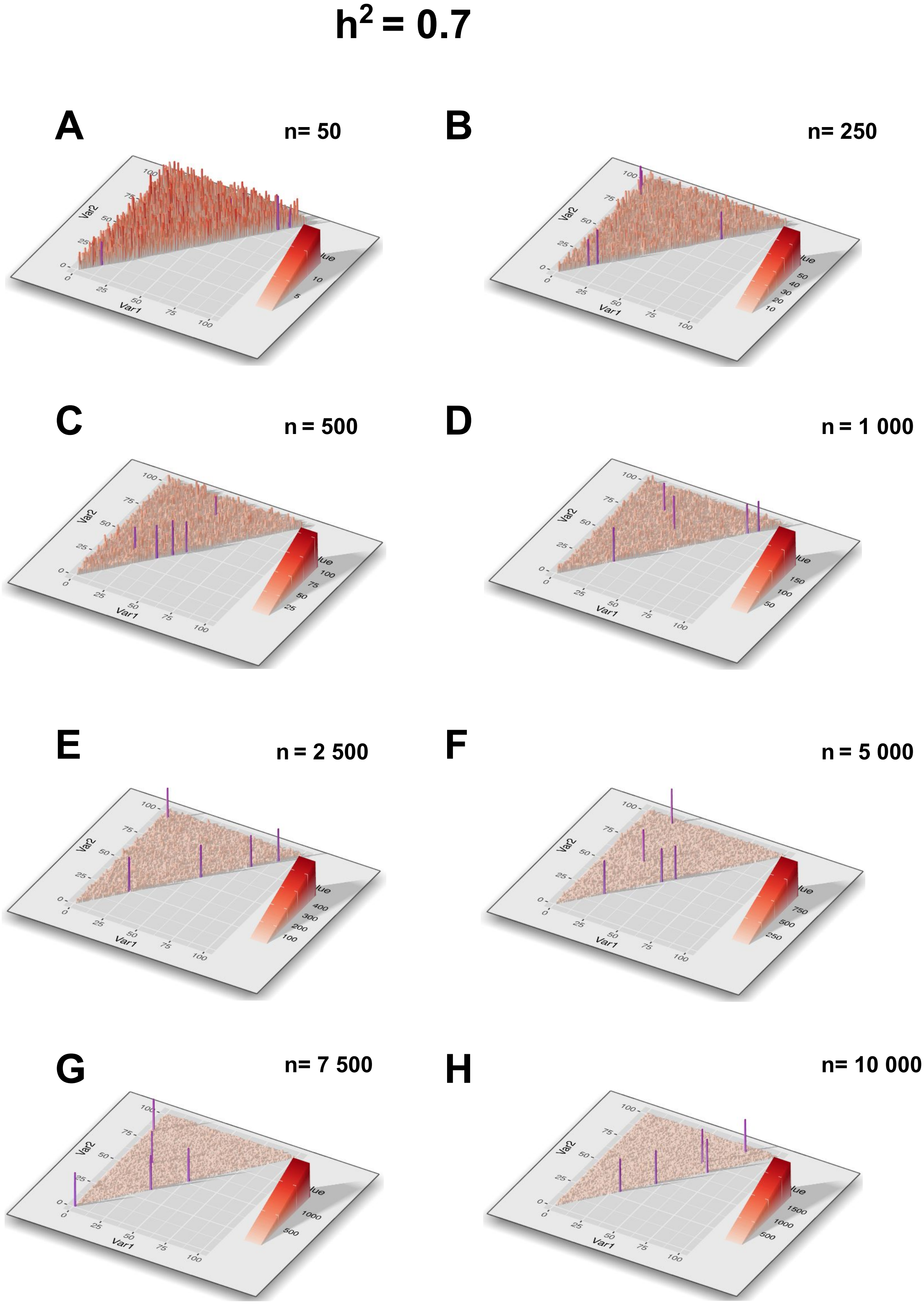

Supplemental Figure 2.

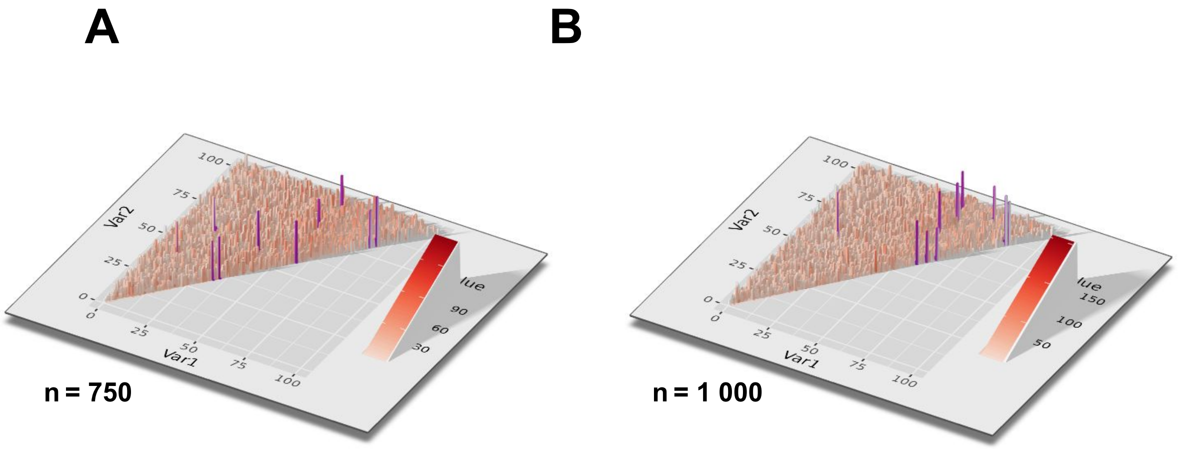

Supplemental Figure 2. Influence of the number of individuals on the power of complex epistatic signal detection. Var1 (x axis) and Var2 (y axis) are a series of 100 SNPs. The triangle corresponds to SNPs combinations when the diagonal contains simple SNPs effects. z axis reports the estimated θ of simple SNPs (diagonal) and combinations (rest of the triangle). Genotype and phenotype data are simulated using specific and modulable parameters (see Github for details and code). Random noise has been added. Simulated signals are in purple. Simulations control heritability h2=0.7. The number of individuals (n) varies over the figures (see details above panels A to B). Four kind of complex epistatic effect have been simulated : 1) simple SNP effect (diagonal only), 2) epistatic interactions but no simple effect (triangle only), 3) true epistatic effect but one of the SNP as a simple effect, 4) SNPs interact with the same SNP having no effect alone. As in Sup Fig 1, purple points mark effects to be reconstructed.

Supplemental Figure 3

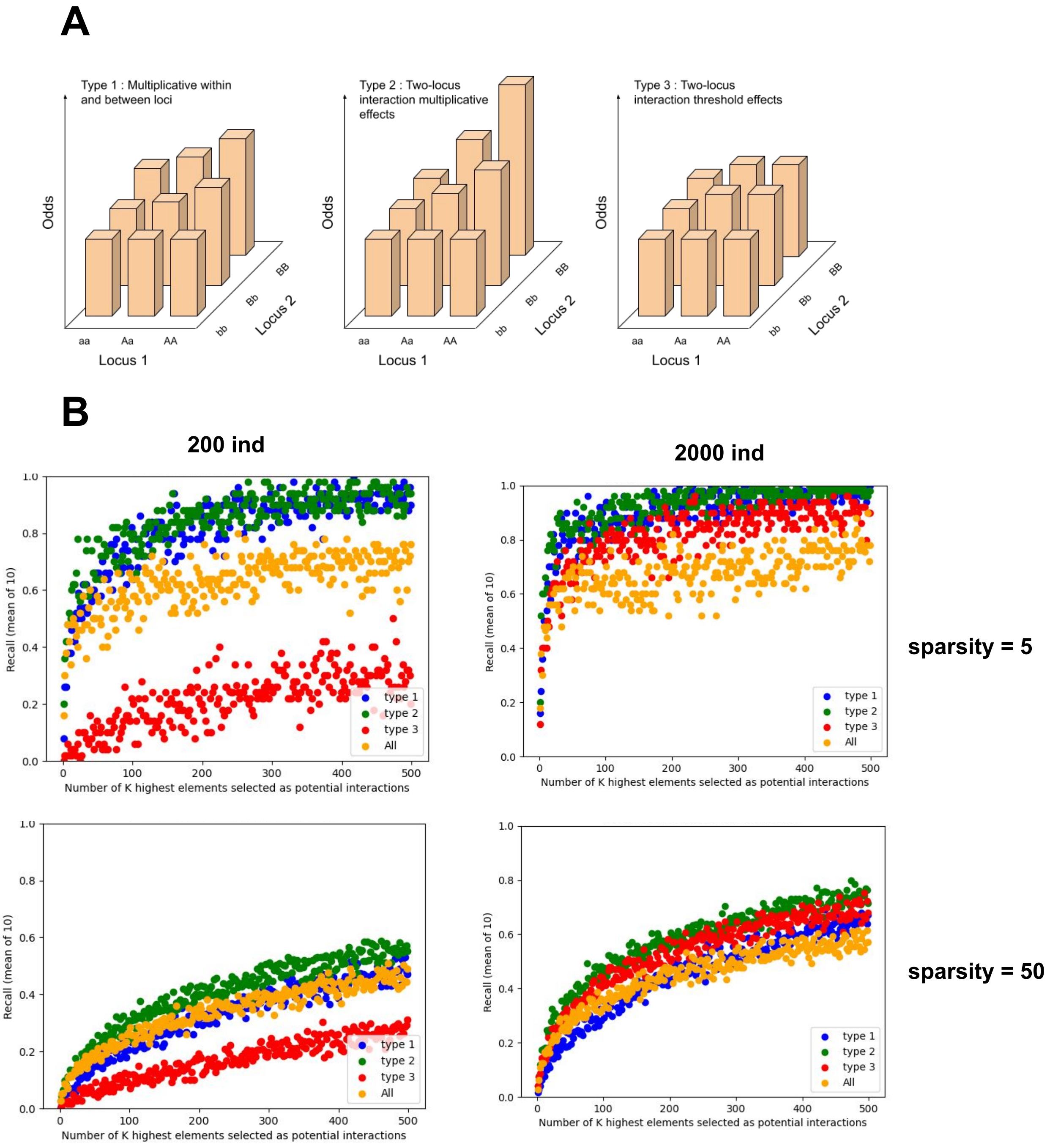

Supplemental Figure 3. Detection capabilities of NGG for 3 types of epistatic interactions.
A) Representation of the 3 types of epistatic interactions that have been simulated following Marchini et al. (2005) procedure. B) Simulation of heterozygous genotypes are performed following the algorithm provided via Github (see Methods). NGG detection algorithm is then used to detect epistatic signals. Plots represent the Recall capabilities of NGG in response to the K top signals (x axis) of NGG for the 3 types of epistatic interactions (type 1, 2, 3, and All [trandom mix of every kind of interactions]). Param: SNP interaction 10000 (as in Sup Fig1), indiv. 200 or 2000, global h2 = 0.5, sparsity 5 or 50.
Value effect for each interaction (theta parameters in Marchini et al. (2005)) randomly set between 0.2 and 0.5 at each draw. Each point correspond to the mean in 10 independent simulations with given parameters.

Supplemental Figure 4.

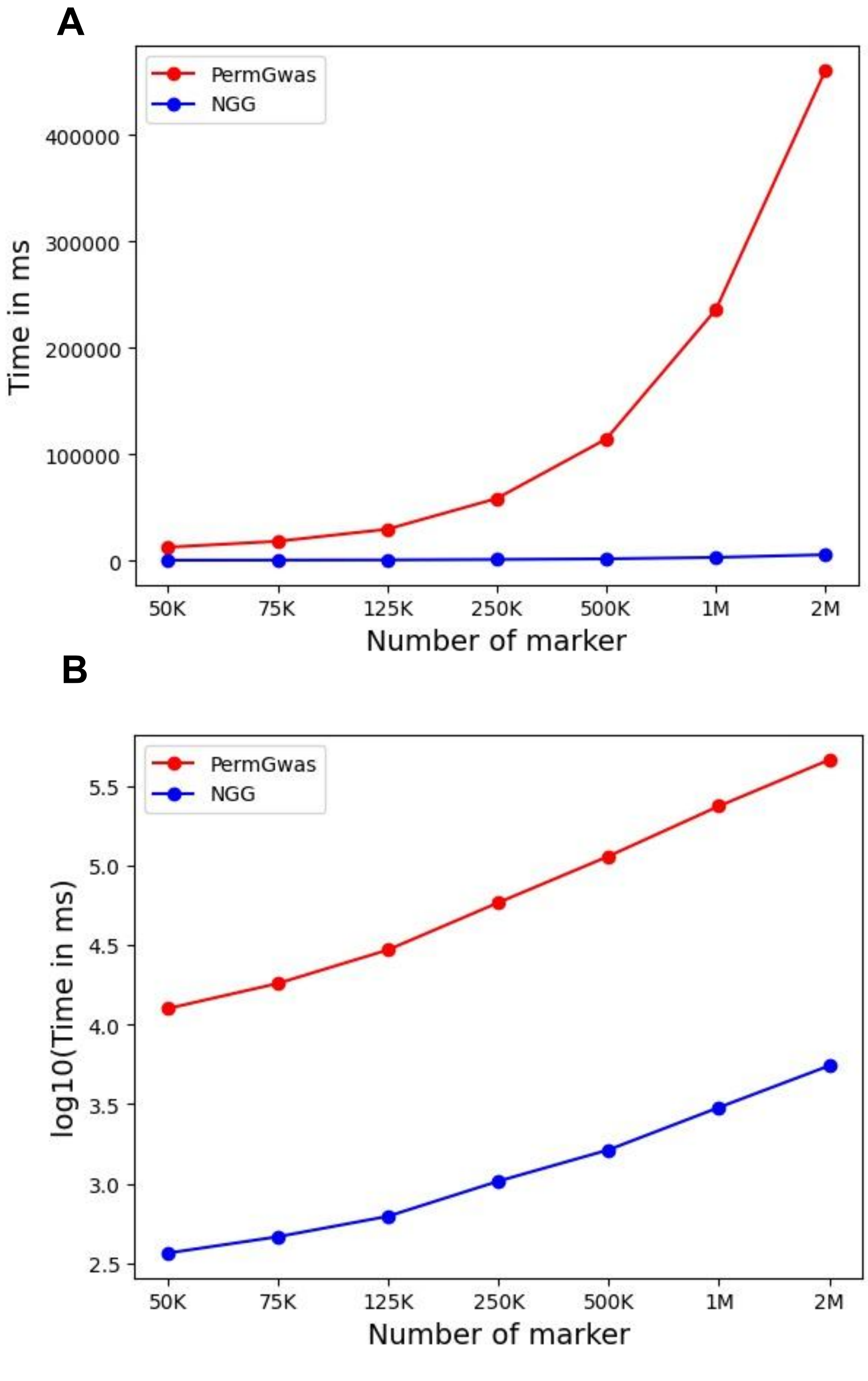

Supplemental Figure 4a. Computation time comparison between permGWAS and NGG as a function of an increasing number of markers. A) Linear scale, B) Log Scale.

Supplemental Figure 4.

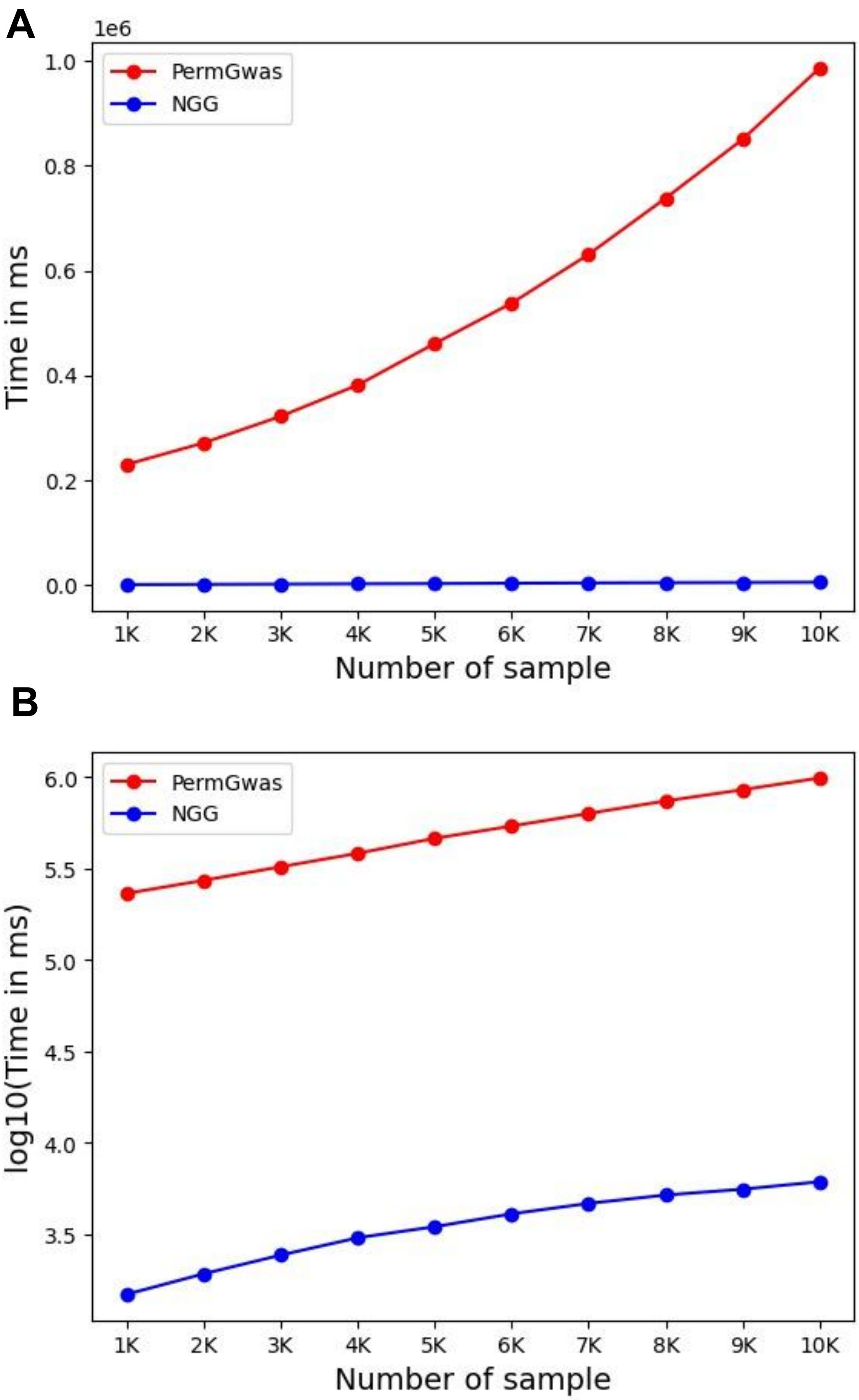

Supplemental Figure 4b. Computation time comparison between permGWAS and NGG as a function of an increasing number of sample. A) Linear scale, B) Log Scale.

Supplemental Figure 5.

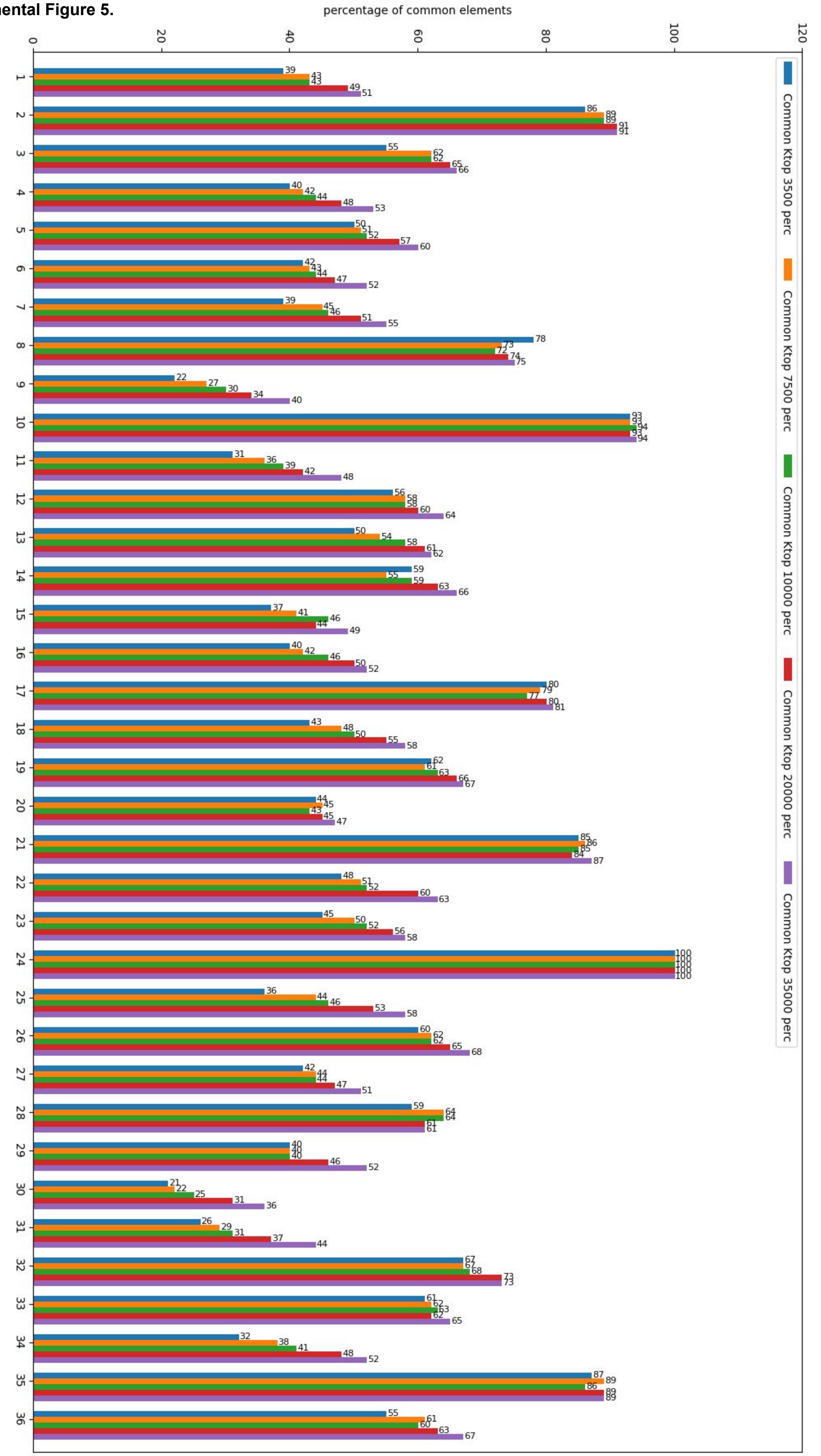

Supplemental Figure 5. Common signal retrieved between EMMA and NGG according to block of Ktop on Atwell et al dataset (phenotype 1 to 36)

Supplemental Figure 5.

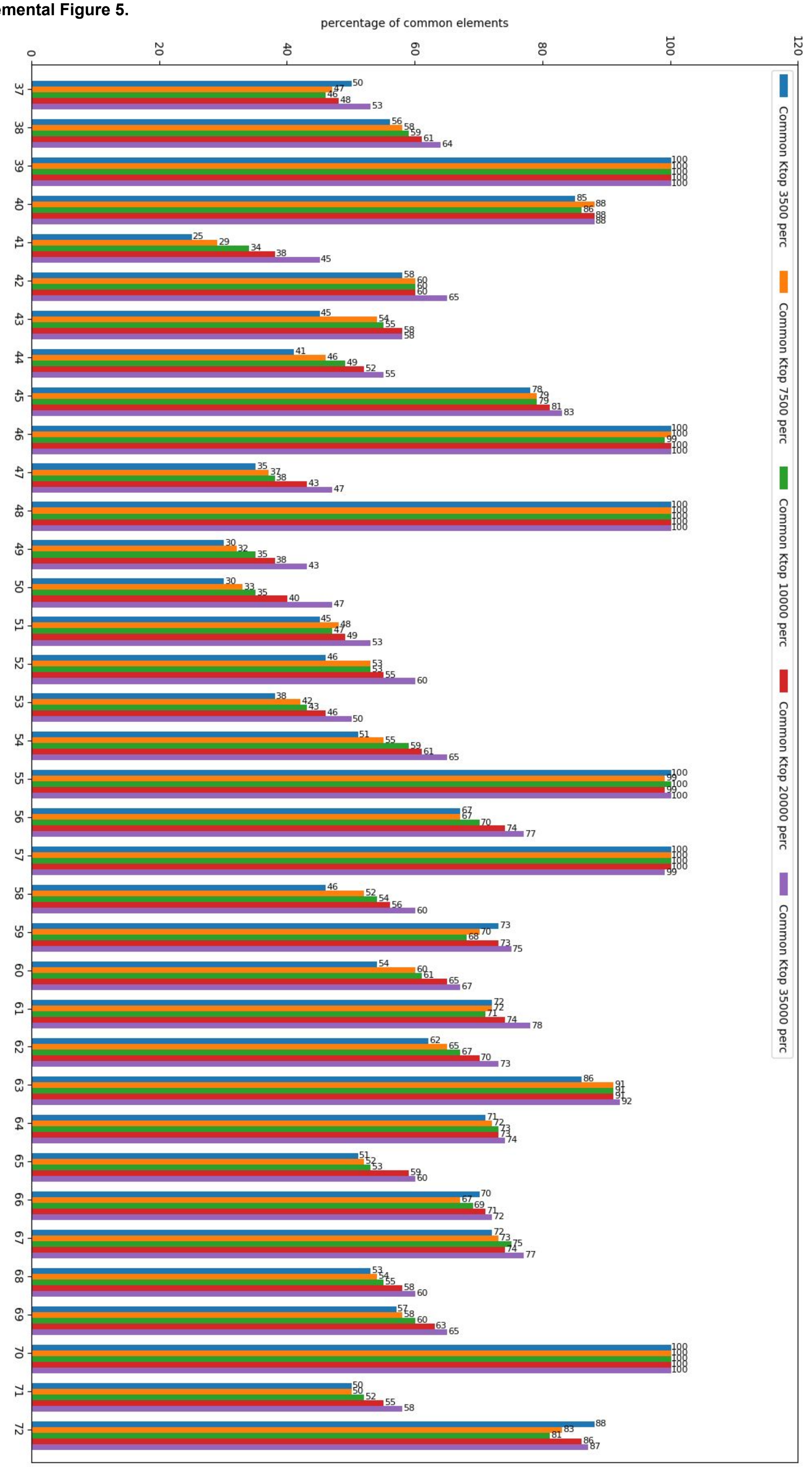

Supplemental Figure 5. Common signal retrieved between EMMA and NGG according to block of Ktop on Atwell et al dataset (phenotype 37 to 72)

Supplemental Figure 5.

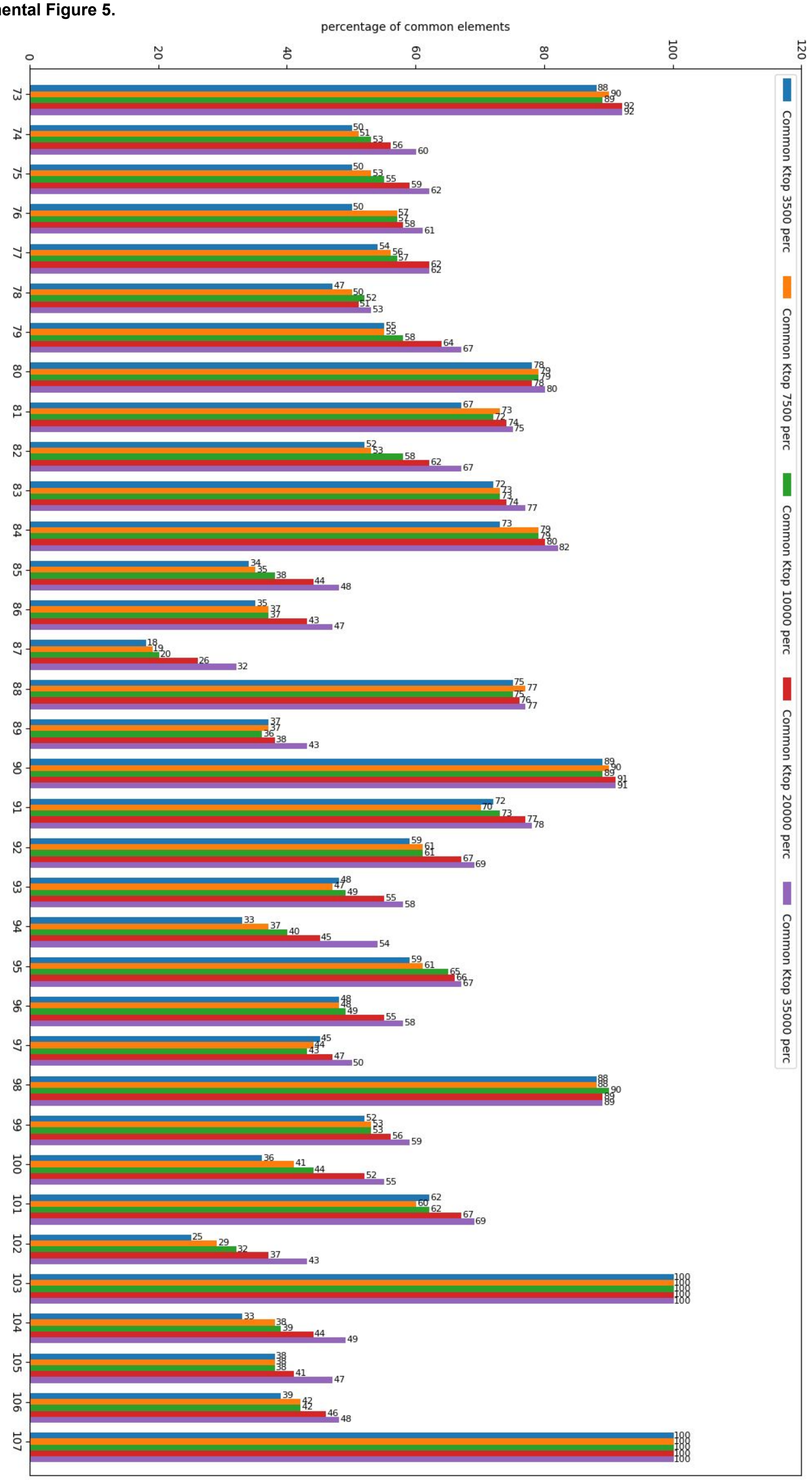

Supplemental Figure 5. Common signal retrieved between EMMA and NGG according to block of Ktop on Atwell et al dataset (phenotype 73 to 107)

Supplemental Figure 5b.

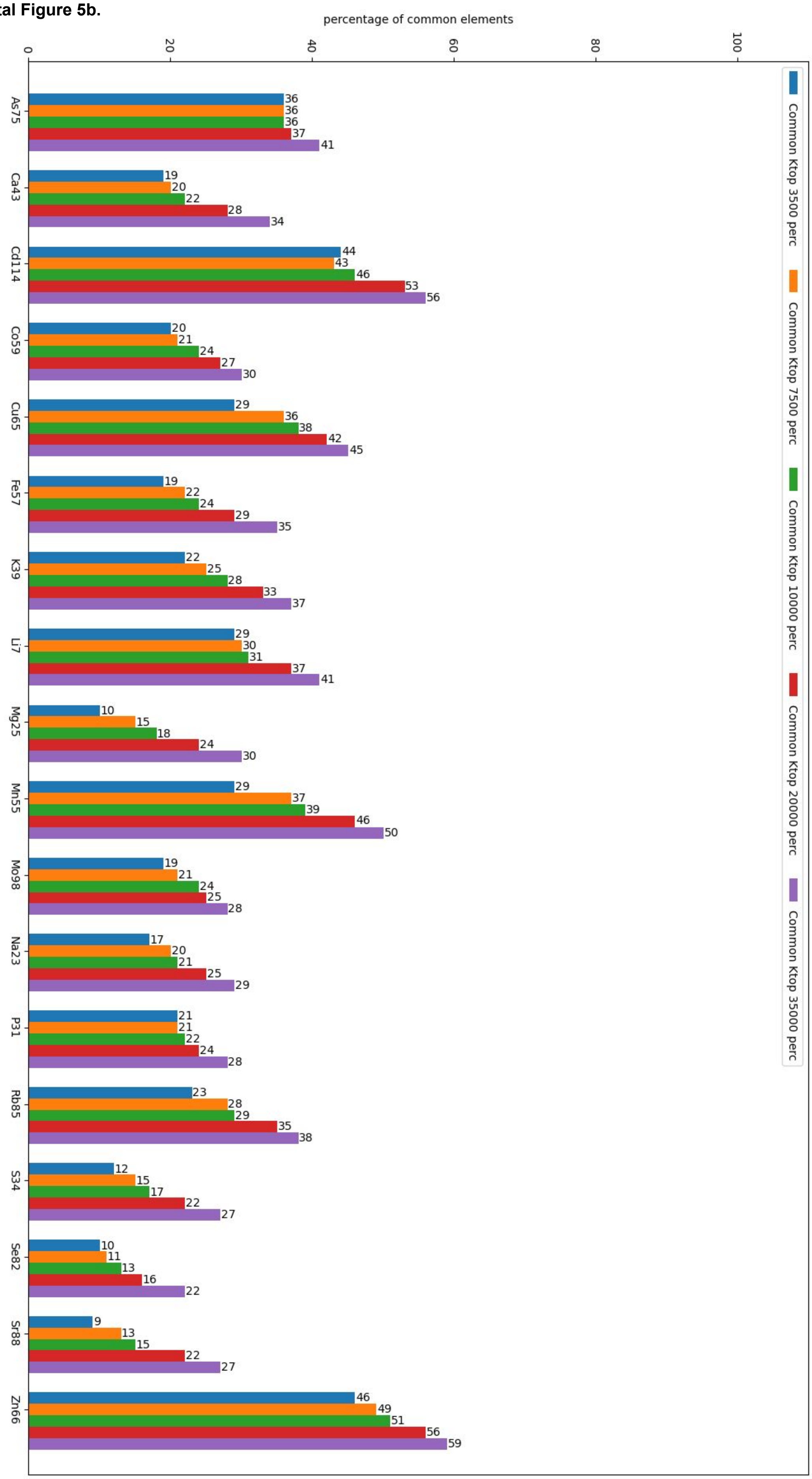

Supplemental Figure 5b. Common signal retrieved between EMMA and NGG according to block of Ktop on Campos et al dataset
