# Supplementary material for: Next-Gen GWAS: full 2D epistatic interaction maps retrieve part of missing heritability and improve phenotypic prediction
Source: Genome Biol. 2024 Mar 25;25:76. doi: 10.1186/s13059-024-03202-0 (PMC10962106; doi:10.1186/s13059-024-03202-0)
Supplement: Supplementary file 1 — Additional file 1. Additional text, figures and table. [file 13059_2024_3202_MOESM1_ESM.zip › Sup Text 1.pdf]

Appendix : NGG algorithm

We start with introducing some notations. Let $\mathcal{E}$ be a set, then $|\mathcal{E}|$ denotes the cardinal of $\mathcal{E}$. If $u = (u_1, \ldots, u_m)$ is a vector of the space $\mathbb{R}^m$ then the support of u is the set of all the non-null coordinates of u :

$$\text{supp}(u) = \{i \in (1, \ldots, m) : u_i \neq 0\}.$$

It is plain that $\text{supp}(u) \subset \{1, \ldots, m\}$. We are ready to define the set of s-sparse vectors :

$$\Theta_{m,s} = \{u \in \mathbb{R}^m : |\text{supp}(u)| = s\}.$$

For further use we will also denote ℓ_k the following space of real sequences :

$$\ell_k = \{(v_n)_{n \in \mathbb{N}} : \Sigma_n |v_n|^k < +\infty\}.$$

We will only mention the spaces ℓ_1 and ℓ_2 within this Appendix.

The algorithm relies on the popular linear regression model:

$$Y = X\theta + \epsilon \tag{1}$$

With $Y \in \mathbb{R}^n$ is the output variable, usually a phenotype and X is the $n \times m$ matrix of inputs collecting genetic information. The j-th column of X displays the data from the j-th SNP. The unknown parameter is $\theta \in \Theta_{m,s}$ and this assumption is crucial. The random variable ϵ stands for a random noise (usually following a Gaussian distribution). The key idea behind the NGG algorithm is as follow:

1. Somewhat "compress the model" (ideally without loss).
2. Solve the compressed model.
3. Recover the original θ from the preceding step.

Depending on the compression/decompression scheme and the resolution algorithm, we could gain in terms of algorithmic complexity. In order to understand the NGG algorithm that is developed in this paper, a brief reminder of the principles of the Compressed Sensing (CS) framework is required ([5], [8]). We start below with the crucial property of Restricted Isometry (RIP).

1 Restricted Isometry Matrices

We give here the definition and -in a nutshell- some tracks in order to construct RIP matrices. The RIP property and the design of RIP matrices are crucial ingredients of the algorithm.

1.1 RIP Property

The Restricted Isometry Property, introduced in ([5]), is as follows: given a matrix $A \in \mathbb{R}^{d\times m}$ and an integer p between 1 and d, A is said to be p-RIP with constant $\delta_p \in (0,1)$ if it exists such δ_p constant for which every $p \times m$ sub-matrix A_p of A satisfy:

$$\forall x \in \Theta_{m,s}, \quad (1-\delta_p)\left\|x\right\|_2^2 \leq \left\|A_p x\right\|_2^2 \leq (1+\delta_p)\left\|x\right\|_2^2 . \tag{2}$$

With other words, any submatrix obtained by extracting p lines from A behaves almost like an isometry. The departure from an exact isometry is measured by the positive δ_p. The important feature is that A maps *approximately* a high-dimensional space $\mathbb{R}^m$ onto a low dimensional space $\mathbb{R}^d$: a reduction dimension is carried out that preserves roughly the distance between the s-sparse points, hence the geometry of a set of s-sparse data.

1.2 Designing RIP Matrices

As one can see in the previous 1.1 section, RIP is not easy to prove for a given matrix, so it rise at least two questions:

1. How can we construct a RIP matrix ?
2. Assuming such an algorithm exists, is it computationally tractable ?

Hopefully, the answer to both questions is positive. In fact, it can be proved ([1], [7]) that -with overwhelming probability- $d \times m$ matrix with either *iid centered Gaussian* or *iid centered Rademacher* entries are RIP enough for compression/decompression with d following $d \sim s \times \log(m)$. It is also known that some matrices with non-random entries are suitable for CS. In [13] for instance the authors propose to subsample the rows of a matrix whose entries are a two-dimensional Discrete Fourier Transform. They compute the associated δ.

2 Sparse recovery

2.1 Exact recovery

Let $\mu \in \Theta_{m,s}$ an s-sparse vector. Let $A \in \mathbb{R}^{d\times m}$ a sensing matrix that satisfies the Restricted Isometry Property (RIP) with $d << m$, typically $d \sim s \times \log(m)$). We call ν the compressed vector of μ from A via the equation:

$$\nu = A\mu. \tag{3}$$

The theory of CS proves that for any s-sparse μ and RIP A with the right constant, the resolution of the following program:

$$\hat{\mu} = \begin{cases} \underset{x\in\mathbb{R}^m}{\operatorname{argmin}} \left\|x\right\|_1 \\ s.t.\ Ax = \nu. \end{cases} \tag{4}$$

yields a $\hat{\mu}$ that is exactly the original μ: i.e. the compression is lossless.

As an example [11] shows that the equation (4) holds for lossless reconstruction with $2s$-RIP matrix A with constant $\delta_{2s} \leq \sqrt{2} - 1$.

In practice, there are multiple ways to solve this optimization program, such as the historical ISTA ([10]) and its improvements FISTA ([3]) and SISTA ([2]) to name a few. Other examples of Basis Pursuit Denoising algorithm are the Dantzig Selector ([6]) or linear program optimization solver such as Primal-Dual ([4]) or HiGHS ([12]))

2.2 Basis Pursuit DeNoising (BPDN)

The basis Pursuit Denoising framework, first introduced in 1994 ([9]) goes a little further and is closely related to the famous LASSO ([14]) in statistics and regression. It focuses on the situation when the compressed vector ν is noisy :

$$\nu = A\mu + \epsilon, \tag{5}$$

where ϵ is unknown and with norm almost surely bounded :

$$\|\epsilon\|_2 \leq \sigma. \tag{6}$$

Then, following ([7]) and with RIP assumption on A, the resolution of the program:

$$\hat{\mu} = \begin{cases} \underset{x \in \mathbb{R}^m}{\operatorname{argmin}} \|x\|_1 \\ s.t.\ \|Ax - \nu\|_2 \leq \sigma \end{cases} \tag{7}$$

gives a $\hat{\mu}$ for which the following oracle inequality holds :

$$\|\mu - \hat{\mu}\|_2 \leq C_0 \|\mu - \mu_s\|_1 + C_1\sigma, \tag{8}$$

where C_0 and C_1 are constants that depend on the δ_{2s} RIP constant of A and μ_s is the best s-sparse representation of μ, thus in the case of a s-sparse μ we have:

$$\|\mu - \hat{\mu}\|_2 \leq C_1\sigma. \tag{9}$$

3 Estimation procedure

With the material of sections 1 and 2 at hand we can turn to describing the statistical aspects of our three-step algorithm. These three steps, already mentioned just after equation (1) are detailed below.

3.1 Compressing the Model

What makes all this works, is the combination of:

- A sound algebraic and simple compression scheme that permits an easy rewriting of the model.
- A compression algorithm in the order of log of the original high dimension, opening the door to the explosive combinatoric nature of the numbers of interactions.

Compressing the θ unknown vector with an RIP-satisfying $A \in \mathbb{R}^{d\times m}$ matrix as

$$\nu = A\theta. \tag{10}$$

We then define

$$A^\dagger = A^T(AA^T)^{-1}. \tag{11}$$

$A^\dagger$ is such that $AA^\dagger = I_{d\times d}$ with $I_{d\times d}$ the identity matrix in $\mathbb{R}^{d\times d}$. We have the following θ decomposition:

$$\theta = A^\dagger\nu + (I_{m\times m} - A^\dagger A)\theta. \tag{12}$$

Now, rewriting model (1) with the decomposition (12), we obtain:

$$Y = XA^\dagger\nu + X(I_{m\times m} - A^\dagger A)\theta + \epsilon. \tag{13}$$

Defining :

$$
\begin{aligned}
W &= XA^{\dagger}, \\
\xi_A &= X(I_{m\times m} - A^{\dagger}A)\theta + \epsilon.
\end{aligned}
$$

The equation (13) can be further simplified as:

$$Y = W\nu + \xi_A. \tag{14}$$

3.2 Solving the Compressed Model

It is of interest to notice that, although the μ vector must be s-sparse for all of this algorithm to works, this is not at all the case for ν. Also, the new noise ξ_A is no more gaussian, nor homoscedastic, introducing some small errors further in the decompressing step. We could have been using (and we tried) some kind of Tikhonov regularization ([15]) for solving the equation (14), but if X is at least of rank d, A being of rank d *by construction*, we have that W is a rank d matrix and the problem is *not* ill-posed. In practice the Ordinary Least Square (OLS) algorithm is enough for a resolution. We write:

$$\widehat{\nu}_A = (W^T W)^{-1} W^T Y. \tag{15}$$

Applying equations (14), (11) and some well known operations on transpositions and inverse of matrices multiplications, equation (15) may be rewritten as :

$$
\begin{aligned}
\widehat{\nu}_A &= ((XA^{\dagger})^T XA^{\dagger})^{-1}(XA^{\dagger})^T Y = (A^{\dagger T} X^T XA^{\dagger})^{-1} A^{\dagger T} X^T Y \\
&= ((A^T(AA^T)^{-1})^T X^T X (A^T(AA^T)^{-1}))^{-1}(A^T(AA^T)^{-1})^T X^T Y \\
&= ((AA^T)^{-1} AX^T XA^T (AA^T)^{-1})^{-1}(AA^T)^{-1} AX^T Y \\
&= (AA^T)(AX^T XA^T)^{-1}(AA^T)(AA^T)^{-1} AX^T Y.
\end{aligned}
$$

Finally, we further simplify as:

$$\widehat{\nu}_A = AA^T(AX^T XA^T)^{-1} AX^T Y. \tag{16}$$

We can see in equation (16) that the inverse matrix problem, usually the costly part, is now about solving a $d \times d$ matrix equation versus a d-length vector, with d in the order of $s \times \log(m)$ for Gaussian sensing matrices. In the case of GWAS2D, m being the number of SNP plus interactions, one can imagine the massive computational advantage this model compression may achieve.

3.3 Recovering θ

The last part is to retrieve $\widehat{\theta}_A$ from $\widehat{\nu}_A$. As stated in 2.1, there is a variety of solvers for the linear problems (4) and (2). In the case at hand, the $\hat{\nu}$ vector is noisy. In fact, if we introduce the "hat matrix" :

$$H_A^X = AA^T(AX^T XA^T)^{-1} AX^T \tag{17}$$

we can rewrite (16) as

$$\widehat{\nu}_A = H_A^X Y = H_A^X X\theta + H_A^X \epsilon. \tag{18}$$

Using equation (12), (14), (17) and (11) successively on θ we obtain:

$$\begin{aligned}
\widehat{\nu}_A &= H_A^X X A^\dagger \nu + H_A^X X (I_{m\times m} - A^\dagger A)\theta + H_A^X \epsilon \\
&= H_A^X X A^\dagger \nu + H_A^X (X (I_{m\times m} - A^\dagger A)\theta + \epsilon) = H_A^X X A^\dagger \nu + H_A^X \xi_A \\
&= AA^T (AX^T X A^T)^{-1} AX^T X A^\dagger \nu + H_A^X \xi_A \\
&= AA^T (AX^T X A^T)^{-1} AX^T X A^T (AA^T)^{-1} \nu + H_A^X \xi_A,
\end{aligned}$$

which is nothing but :

$$\widehat{\nu}_A = \nu + H_A^X \xi_A. \tag{19}$$

So the problem at hand becomes: can we find θ when the measured compressed vector $\hat{\nu}$ is a noisy version of ν ? As seen in 2.2, the answer is yes, under certain conditions on the noise $H_A^X \xi_A$. Using equations (18) and (10), we can rewrite the equation (19) as:

$$\begin{aligned}
\widehat{\nu}_A &= \nu + H_A^X \xi_A = A\theta + H_A^X \xi_A, \\
H_A^X Y &= A\theta + H_A^X \xi_A.
\end{aligned}$$

Which brings us back to equation (5) that we can solve with the resolution of:

$$\hat{\theta} = \begin{cases} \underset{x\in\mathbb{R}^m}{\operatorname{argmin}} \|x\|_1, \\ s.t. \ \left\|Ax - H_A^X Y\right\|_2 \leq \sigma_{H_A^X \xi_A}, \end{cases} \tag{20}$$

where $\sigma_{H_A^X \xi_A}$ is such that $\left\|H_A^X \xi_A\right\|_2 \leq \sigma_{H_A^X \xi_A}$.

4 Algorithm acceleration

One major caveat with the approach detailed above is that the recovery algorithm may be very costly. In fact, the algorithm presented in section 3 is faster than the standard approach but not so much as expected for very high-dimensional datasets. In this section, we discuss an improvement of the NGG algorithm in terms of computation time. This acceleration is carried out with a slight damage on the precision.

4.1 Sub-Sampling Scheme

The choice of d has not been addressed yet. This value is important for the whole algorithm to function cleanly. The CS theory claims that d should be greater than $C \times s \times \log(m)$ with C a constant that does not depend on X and Y. It is important to recall that s is *unknown*, so we do not have a standard routine to select an adaptive d.

Another concern is the inversion of the $d \times d$ matrix $AX^T X A^T$. This means that matrix X must be at least of rank d. In other words we must choose a d that is *at most* the rank of X, if X is $n \times m$, with $n << m$, d must be lower than n (the number of accessions).

Validating both constraints is not always possible. Hopefully, it happens that for low values of d, the algorithm still performs nicely. A the strong signal will be detected at the cost of losing weaker signals. We are aware of this limitation in the context of SNP interactions. Usually, interactions terms are thought to be of weaker signal value than 0th-order signals, in practice, we still find sound and coherent signals in the interaction terms and additional work is to be done to try to upscale this algorithm recall for true values.

4.2 Piecewise Solving

We introduce another trick allowing a better parallelization of the algorithm.

For generalization, start with a random permutation τ of the columns of X and rows of θ and choose a number q of first elements. Following the classic Python way of writing array slices, we denote ": q" for the first q elements and "q :" for the remaining ($m-q$ here) elements. We can write the model (1) as:

$$Y = \begin{pmatrix} X_{\tau|:q} & X_{\tau|q:} \end{pmatrix} \begin{pmatrix} \theta_{\tau|:q} \\ \theta_{\tau|q:} \end{pmatrix} + \epsilon. \tag{21}$$

We now choose A_q, an $d \times q$ RIP matrix, write:

$$A = \begin{pmatrix} A_q & 0_{d\times(m-q)} \end{pmatrix} \tag{22}$$

and rewind all the preceding calculations using the decompositions (21) and (22). First we rewrite the isometric transformation ν of θ:

$$\nu \;=\; A\theta = \begin{pmatrix} A_q & 0_{d\times(m-q)} \end{pmatrix} \begin{pmatrix} \theta_{\tau|:q} \\ \theta_{\tau|q:} \end{pmatrix} = A_q \theta_{\tau|:q}. \tag{23}$$

Therefore, we have the following decomposition for $A^\dagger$:

$$\begin{aligned} A^\dagger &= \begin{pmatrix} A_q^T \\ 0_{(m-q)\times d} \end{pmatrix} \left(\begin{pmatrix} A_q & 0_{d\times(m-q)} \end{pmatrix} \begin{pmatrix} A_q^T \\ 0_{(m-q)\times d} \end{pmatrix} \right)^{-1} \\ &= \begin{pmatrix} A_q^T \\ 0_{(m-q)\times d} \end{pmatrix} \left(A_q A_q^T + 0_{d\times(m-q)} 0_{(m-q)\times d} \right)^{-1} \\ &= \begin{pmatrix} A_q^\dagger \\ 0_{(m-q)\times d} \end{pmatrix}. \end{aligned}$$

Then it is now the turn for θ to be rewritten:

$$\begin{aligned} \theta &= A^\dagger \nu + (I_{m\times m} - A^\dagger A)\theta \\ &= \begin{pmatrix} A_q^\dagger \\ 0_{(m-q)\times d} \end{pmatrix} \nu + \left(I_{m\times m} - \begin{pmatrix} A_q^\dagger \\ 0_{(m-q)\times d} \end{pmatrix} \begin{pmatrix} A_q & 0_{d\times(m-q)} \end{pmatrix} \right) \theta \\ &= \begin{pmatrix} A_q^\dagger \nu \\ 0_{(m-q)\times d} \end{pmatrix} + \begin{pmatrix} I - A_q^\dagger A_q & 0 \\ 0 & I \end{pmatrix} \begin{pmatrix} \theta_{\tau|:q} \\ \theta_{\tau|q:} \end{pmatrix} \end{aligned}$$

which gives us the following θ decomposition:

$$\theta = \begin{pmatrix} A_q^\dagger \nu + \left(I_{q\times q} - A_q^\dagger A_q \right) \theta_{\tau|:q} \\ \theta_{\tau|q:} \end{pmatrix}. \tag{24}$$

Going back to the base model (1):

$$\begin{aligned} Y &= X\theta + \epsilon \\ &\overset{by\ (21)+(24)}{=} \begin{pmatrix} X_{\tau|:q} & X_{\tau|q:} \end{pmatrix} \begin{pmatrix} A_q^\dagger \nu + \left(I_{q\times q} - A_q^\dagger A_q \right) \theta_{\tau|:q} \\ \theta_{\tau|q:} \end{pmatrix} + \epsilon \\ &= X_{\tau|:q} A_q^\dagger \nu + X_{\tau|:q} \left(I_{q\times q} - A_q^\dagger A_q \right) \theta_{\tau|:q} + X_{\tau|q:} \theta_{\tau|q:} + \epsilon. \end{aligned}$$

Let us turn to H_A^X and rewrite it like above, using the fact that $AA^T = A_q A_q^T$ and that:

$$\begin{aligned}
AX^T &= \begin{pmatrix} A_q & 0_{d\times(m-q)} \end{pmatrix} \begin{pmatrix} X_{\tau|:q}^T \\ X_{\tau|q:}^T \end{pmatrix} \\
&= A_q X_{\tau|:q}^T, \\
AX^T X A^T &= \begin{pmatrix} A_q & 0_{d\times(m-q)} \end{pmatrix} \begin{pmatrix} X_{\tau|:q}^T \\ X_{\tau|q:}^T \end{pmatrix} \begin{pmatrix} X_{\tau|:q} & X_{\tau|q:} \end{pmatrix} \begin{pmatrix} A_q^T \\ 0_{(m-q)\times d} \end{pmatrix} \\
&= A_q X_{\tau|:q}^T X_{\tau|:q} A_q^T.
\end{aligned}$$

we obtain the following equation for H_A^X:

$$H_A^X = AA^T(AX^TXA^T)^{-1}AX^T \tag{25}$$

$$= A_q A_q^T (A_q X_{\tau|:q}^T X_{\tau|:q} A_q^T)^{-1} A_q X_{\tau|:q}^T \equiv H_{A_q}^{X_{\tau|:q}} \tag{26}$$

then

$$H_A^X Y = H_A^X X_{\tau|:q} A_q^\dagger \nu + H_A^X X_{\tau|:q} \left(I_{q\times q} - A_q^\dagger A_q\right) \theta_{\tau|:q} + H_A^X X_{\tau|q:} \theta_{\tau|q:} + H_A^X \epsilon \tag{27}$$

where

$$\begin{aligned}
H_A^X X_{\tau|:q} A_q^\dagger \nu &= A_q A_q^T (A_q X_{\tau|:q}^T X_{\tau|:q} A_q^T)^{-1} A_q X_{\tau|:q}^T X_{\tau|:q} A_q^\dagger \nu \\
&= A_q A_q^T (A_q X_{\tau|:q}^T X_{\tau|:q} A_q^T)^{-1} A_q X_{\tau|:q}^T X_{\tau|:q} A_q^T \left(A_q A_q^T\right)^{-1} \nu \\
&= \nu,
\end{aligned}$$

and

$$H_A^X X_{\tau|:q} \left(I_{q\times q} - A_q^\dagger A_q\right) \theta_{\tau|:q} + H_A^X X_{\tau|q:} \theta_{\tau|q:} = H_A^X X \begin{pmatrix} I - A_q^\dagger A_q & 0 \\ 0 & I \end{pmatrix} \theta.$$

At last we have :

$$H_A^X Y = \nu + H_A^X \left(X \begin{pmatrix} I - A_q^\dagger A_q & 0 \\ 0 & I \end{pmatrix} \theta + \epsilon \right). \tag{28}$$

We now define ξ_A^q as:

$$\xi_A^q = X \begin{pmatrix} I - A_q^\dagger A_q & 0 \\ 0 & I \end{pmatrix} \theta + \epsilon$$

and equation (28) becomes :

$$\begin{aligned}
H_A^X Y &= \nu + H_A^X \xi_A^q \\
H_{A_q}^{X_{\tau|:q}} Y &\overset{by\ (26)+(23)}{=} A_q \theta_{\tau|:q} + H_{A_q}^{X_{\tau|:q}} \xi_A^q.
\end{aligned}$$

The decompression equations to solve (20) are now:

$$\widehat{\theta}_{\tau|:q} = \begin{cases} \underset{x\in\mathbb{R}^q}{\operatorname{argmin}} \|x\|_1 \\ s.t.\ \left\| A_q x - H_{A_q}^{X_{\tau|:q}} Y \right\|_2 \le \sigma_{H_{A_q}^{X_{\tau|:q}} \xi_A^q}. \end{cases} \tag{29}$$

This shows that we are able to estimate chosen parts of θ with essentially the same algorithm applied to the corresponding sub-section of columns of X and we can have an idea of the price

of doing so via equation (29) by studying the fluctuations of the value of $\left\|H^{X_{\tau|:q}}_{A_q}\xi^q_A\right\|_2$. Going back to equation (27) we have:

$$H^{X_{\tau|:q}}_{A_q}\xi^q_A = H^X_A X_{\tau|:q}\left(I_{q\times q} - A^\dagger_q A_q\right)\theta_{\tau|:q} + H^X_A X_{\tau|q:}\theta_{\tau|q:} + H^X_A \epsilon.$$

We can deconstruct this noise into three parts:

- The first part $H^X_A X_{\tau|:q}\left(I_{q\times q} - A^\dagger_q A_q\right)\theta_{\tau|:q}$ is the "attenuation" of the θ part we are interested in, this is what brings the existing signal in the ν part. This part depends on X and θ only via their first q elements.

- The second part $H^X_A X_{\tau|q:}\theta_{\tau|q:}$ is the remaining $m-q$ columns of X. It is but sphericized [André...] via the $H^{X_{\tau|:q}}_{A_q}$ operator that only depends on the first q columns of X. This part depends fully on X but only on the $m-q$ remaining elements of θ.

- The third part $H^X_A\epsilon$ is about the original noise ϵ which is going through the $H^{X_{\tau|:q}}_{A_q}$ operator, which does not depend on θ.

At this point, it may be useful to recall that it is assumed that θ is s-sparse, which leads to three cases:

1. The majority of the s non-null elements are in $\theta_{\tau|:q}$, the first part attenuate this and the signal is therefore in ν, ready to be found. The second part is mostly zeros, and as such its ℓ_2 norm is low.

2. The majority of the s non-null elements are in $\theta_{\tau|q:}$, the first part is near zero in term of ℓ_2 norm, and the remaining noise is high, the estimation will be near zero, *as it should be.*

3. The signal is approximately separated between $\theta_{\tau|:q}$ and $\theta_{\tau|q:}$. Nothing could be said *a priori* about what the $\widehat{\theta}_{\tau|:q}$ is likely to recover. In practice, we found that, although it did fail to recover all of the interesting coordinates of $\theta_{\tau|:q}$, it managed to find *some* of them (usually the most prominent part).

4.3 Averaging and Support

A way we can palliate the problem of the last point of 4.2 is via a simple rerun and averaging algorithm. Unlike the classic Compressed Sensing scenario where we sample a signal on the run, in this case we can apply the recipe *ad nauseam* with new simulated A matrices. Take the case of one iteration of a multiple reruns with new indices τ and compression matrix A, if we are in the case of points 1. and 2. of 4.2, high signal coordinates are retrieved and low signal is truthfully evicted. The problem arise with point 3. where one or many of the θ values associated with the indices can be miss-computed (high for low and low for high), in this case, when running the algorithm again, this faulty indices will be again in one of the 3 cases, and if they fall in case 1. or 2. their values will be "corrected". If they fall in the case 3. again, their is always another chance for them to be chosen again, hopefully in good conditions for a good estimation. Of course, there is a price to pay in terms of accuracy and shrinkage, but as often in GWAS we are more interested in finding the support of the unknown slope parameter θ vector more than estimating its coordinates with precision.

4.4 The Final Algorithm

In order to write the full NGG algorithm, we need to set up a few functions that are parts of the global algorithm and subject to customization.

4.4.1 Partitioning

The following algorithm is designed to generate partitions of $\{1, ..., M\}$, without replacement, in order for NGG to see all SNPs and interactions during the next phases. One of the simplest partition algorithm is just to generate blocs of q indices after shuffling the list of indices.

Algorithm 1 shuffle & cut Partitioning

```
function PARTITION(M, q)
    σ ← {1, ..., M}
    shuffle σ
    Σ ← ∅
    for i = 0 to ⌊(M − 1)/q⌋ do
        Σ ← Σ ∪ σ_|(i*q+1):((i+1)*q+1)
    end for
    Σ ← Σ ∪ σ_(⌊(M−1)/q⌋*q+1):M                ▷ In case q and M are co-primes
    return Σ
end function
```

Another possibility could be to simply generate random partitions of size q and hopping that for a great number of iterations we will visit each SNPs and interactions a sufficient number of times. This lead us to a modified NGG algorithm (algorithm 6) where we lose some control over the fidelity of reconstruction but with the benefice of greater parallelism opportunities.

Algorithm 2 Random Partitioning

```
function RANDOMPARTITION(M, q)
    require: RAND(min, max) an integer-valued uniform random generator.
    σ ← ∅
    while |σ| < q do
        r ← RAND(1, M)
        while r ∈ σ do
            r ← RAND(1, M)
        end while
        σ ← σ ∪ r
    end while
    return σ
end function
```

4.4.2 RIP matrices

The second point of customization is the RIP-matrix-generator. In our case, we will go with the simple Gaussian-entry algorithm:

Algorithm 3 Gaussian RIP matrices generator

```
function RIPMATRIX(d, q)
    require: RANDN(mean, stdvar) a real-valued Gaussian random generator.
    A ← 0 ∈ ℝ^{d×q}
    for i = 1 to d do
        for j = 1 to q do
            A[i, j] = RANDN(0, 1)
        end for
    end for
    return A
end function
```

4.4.3 BPDN

The third and last point of customization is the *BPDN* solving algorithm. As previously stated, there is a plethora of algorithms that can be used to solve this exact problem. We choose to go with the FISTA [3] algorithm:

Algorithm 4 FISTA (Simplified pseudo code)

```
function SHRINKAGE(α, x)        ▷ Every operations are applied coordinate by coordinate.
    return (|x| − α)_+ SIGN(x)
end function

function BPDN(A, ν)
    require: A ∈ ℝ^{d×q}
    require: A is RIP.
    require: ν ∈ ℝ^d
    require: λ ← a value to be computed accordingly.        ▷ See [3] for details.
    require: ϵ a low error value.
    x_0 ← 0 ∈ ℝ^q        ▷ First guess.
    y_1 ← x_0
    t_1 ← 1
    k ← 0
    repeat
        k ← k + 1
        x_k ← SHRINKAGE(λ, y_k − 2tA^T(Ay_k − b))
        t_{k+1} ← (1 + sqrt(1 + 4t_k^2)) / 2
        y_{k+1} ← x_k + ((t_k − 1) / t_{k+1}) (x_k − x_{k−1})
    until ||x_k − x_{k−1}|| ≤ ϵ        ▷ Can be a number of iterations criterion.
    return x_k
end function
```

4.4.4 NGG algorithm

At long last, we can now write the full NGG algorithm:

Algorithm 5 NGG (full partitioning)

```
function NGG(X, Y, d, iter)
    require: X ∈ {0,1,2}^{n×m}
    require: Y ∈ ℝ^n
    require: m >> n
    require: d ≤ Rank(X)
    require: iter ≥ 1
    M ← m(m+1)/2
    Z ← [X X·X]                                   ▷ Z is the full X and interactions
    θ* ← 0 ∈ ℝ^M                                  ▷ Result vector
    for i = 1 to iter do
        Σ ← Partition(M, q)
        for all σ ∈ Σ do                          ▷ Easily parallel.
            A_σ ← RIPmatrix(d, q)
            Z_|σ ← the sub sample of columns of Z of indices in σ.
            H_σ ← A_σ A_σ^T (A_σ Z_|σ^T Z_|σ A_σ^T)^{-1} A_σ Z_|σ^T
            θ*_|σ ← θ*_|σ + BPDN(A_σ, H_σ Y)
        end for
    end for
    return θ*/iter
end function
```

And its random partitioning variant:

Algorithm 6 NGG (random partitioning)

```
function NGG(X, Y, d, iter)
    require: X ∈ {0,1,2}^{n×m}
    require: Y ∈ ℝ^n
    require: m >> n
    require: d ≤ Rank(X)
    require: iter ≥ 1
    M ← m(m+1)/2
    Z ← [X X·X]                                   ▷ Z is the full X and interactions
    θ* ← 0 ∈ ℝ^M                                  ▷ Result vector
    N* ← 0 ∈ ℕ^M                                  ▷ Count vector
    for i = 1 to iter do                          ▷ Further parallelization.
        σ ← RandomPartition(M, q)
        A_σ ← RIPmatrix(d, q)
        Z_|σ ← the sub sample of columns of Z of indices in σ.
        H_σ ← A_σ A_σ^T (A_σ Z_|σ^T Z_|σ A_σ^T)^{-1} A_σ Z_|σ^T
        θ*_|σ ← θ*_|σ + BPDN(A_σ, H_σ Y)
        N*_|σ ← N*_|σ + 1                         ▷ 1 is the vector of ones in ℕ^q
    end for
    return θ*./N*                                 ▷ ./ is the piece-wise division
end function
```

References

[1] Richard Baraniuk, Mark Davenport, Ronald DeVore, and Michael Wakin. A simple proof of the restricted isometry property for random matrices. *Constructive Approximation*, 28(3):253–263, January 2008.

[2] Ilker Bayram and Ivan W. Selesnick. A subband adaptive iterative shrinkage/thresholding algorithm. *IEEE Transactions on Signal Processing*, 58(3):1131–1143, 2010.

[3] Amir Beck and Marc Teboulle. A fast iterative shrinkage-thresholding algorithm for linear inverse problems. *SIAM J. Imaging Sci.*, 2(1):183–202, January 2009.

[4] C. Brauer, D. A. Lorenz, and A. M. Tillmann. A Primal-Dual Homotopy Algorithm for ℓ_1-Minimization with ℓ_∞-Constraints. *Computational Optimization and Applications*, 70(2):443–478, 2018. DOI:10.1007/s10589-018-9983-4.

[5] E.J. Candes and T. Tao. Decoding by linear programming. *IEEE Transactions on Information Theory*, 51(12):4203–4215, 2005.

[6] Emmanuel Candes and Terence Tao. The Dantzig selector: Statistical estimation when p is much larger than n. *The Annals of Statistics*, 35(6):2313 – 2351, 2007.

[7] Emmanuel J Candès. The restricted isometry property and its implications for compressed sensing. *C. R. Math. Acad. Sci. Paris*, 346(9-10):589–592, May 2008.

[8] Emmanuel J. Candes and Terence Tao. Near-optimal signal recovery from random projections: Universal encoding strategies? *IEEE Transactions on Information Theory*, 52(12):5406–5425, 2006.

[9] Shaobing Chen and D. Donoho. Basis pursuit. In *Proceedings of 1994 28th Asilomar Conference on Signals, Systems and Computers*, volume 1, pages 41–44 vol.1, 1994.

[10] I Daubechies, M Defrise, and C De Mol. An iterative thresholding algorithm for linear inverse problems with a sparsity constraint. *Commun. Pure Appl. Math.*, 57(11):1413–1457, November 2004.

[11] D.L. Donoho. Compressed sensing. *IEEE Transactions on Information Theory*, 52(4):1289–1306, 2006.

[12] Qi Huangfu and Julian A. J. Hall. Parallelizing the dual revised simplex method. *Mathematical Programming Computation volume*, 10:119–142, 2018.

[13] Mark Rudelson and Roman Vershynin. On sparse reconstruction from fourier and gaussian measurements. *Communications on Pure and Applied Mathematics*, 61(8):1025–1045, 2008.

[14] Robert Tibshirani. Regression shrinkage and selection via the lasso. *Journal of the Royal Statistical Society: Series B (Methodological)*, 58(1):267–288, 1996.

[15] A. N. Tikhonov and Arsenin V. Y. *Solutions of ill-posed problems / Andrey N. Tikhonov and Vasiliy Y. Arsenin ; translation editor, Fritz John.* Scripta series in mathematics. Winston ; New York : distributed solely by Halsted Press, Washington, 1977.
