# Supplementary figures and images for: Next-Gen GWAS: full 2D epistatic interaction maps retrieve part of missing heritability and improve phenotypic prediction
Source: Genome Biol. 2024 Mar 25;25:76. doi: 10.1186/s13059-024-03202-0 (PMC10962106; doi:10.1186/s13059-024-03202-0)

As75

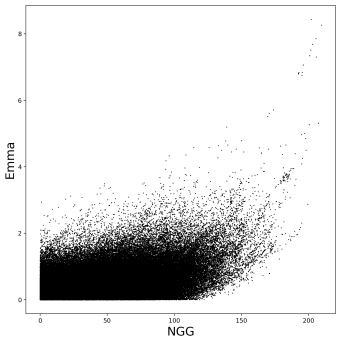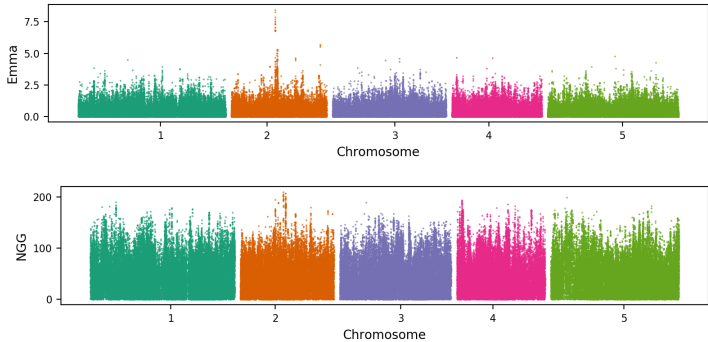

Ca43

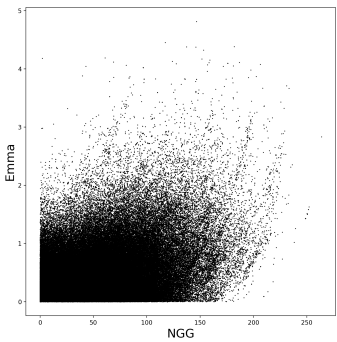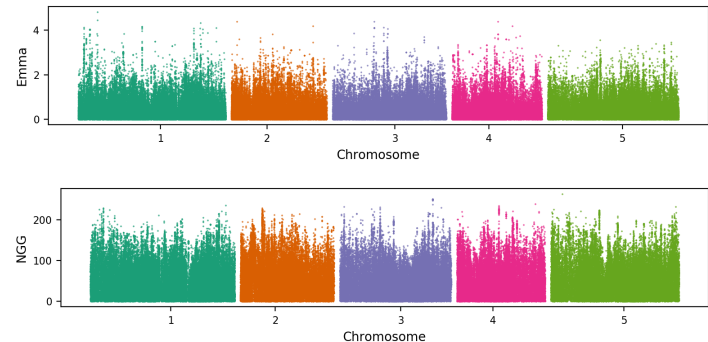

Cd114

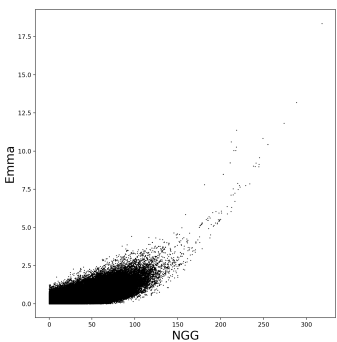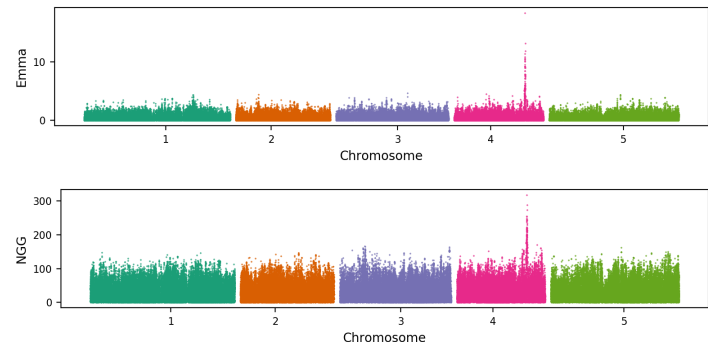

Co59

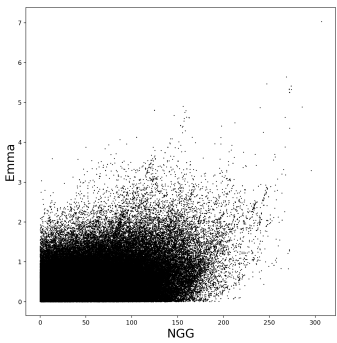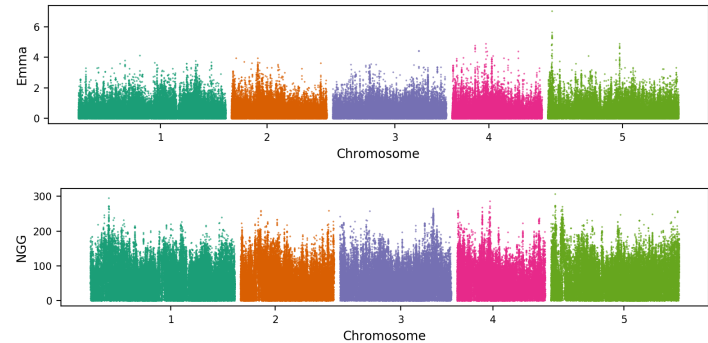

Cu65

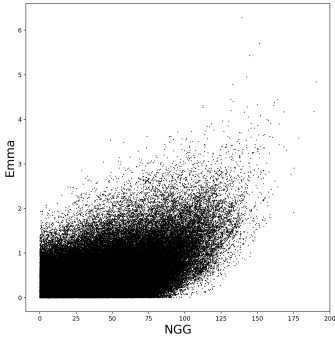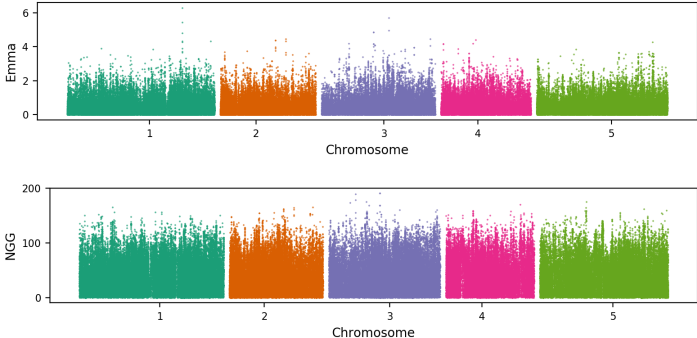

Fe57

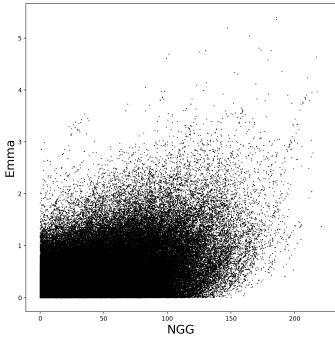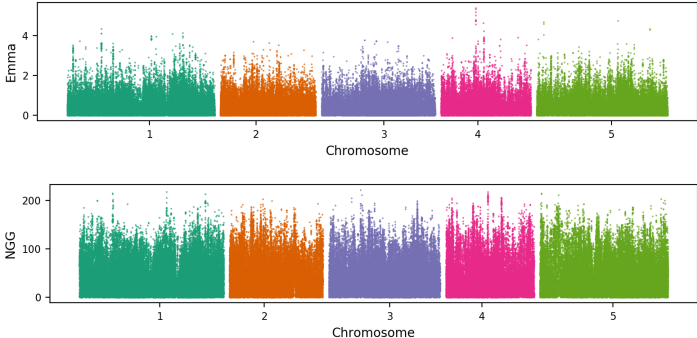

K39

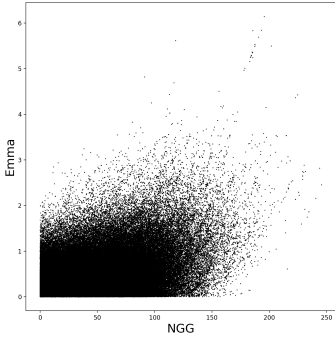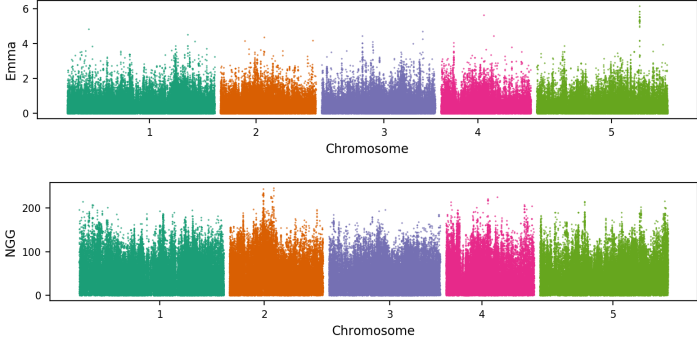

Li7

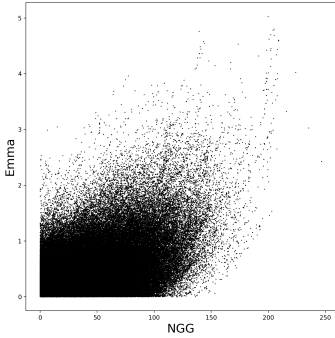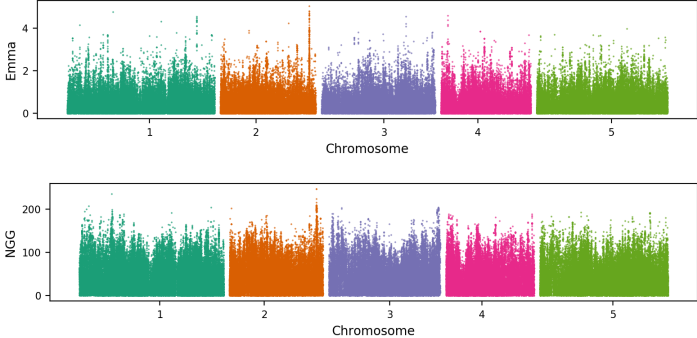

Mg25

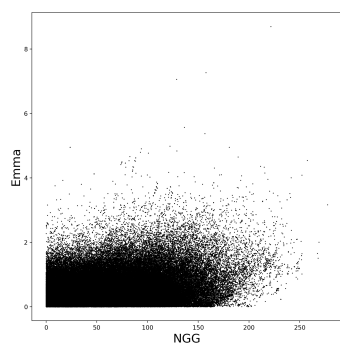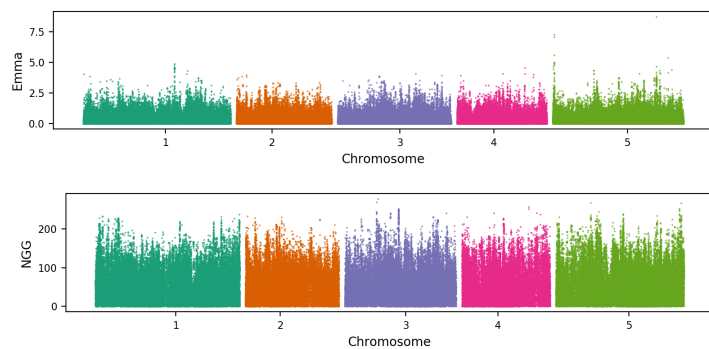

Mn55

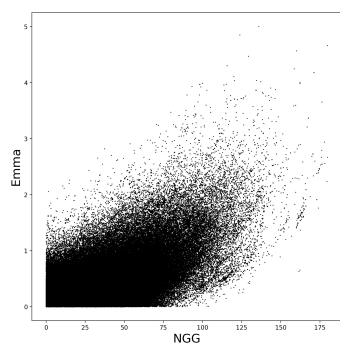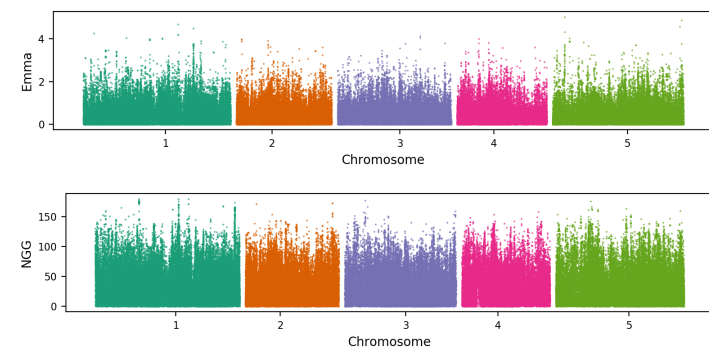

Mo98

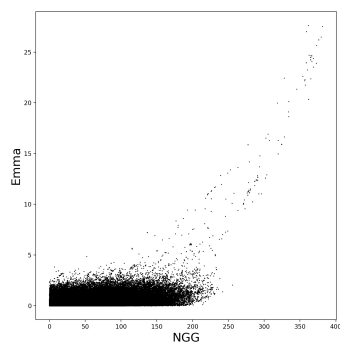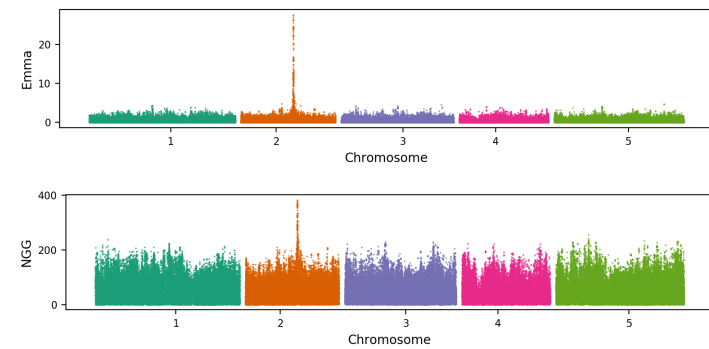

Na23

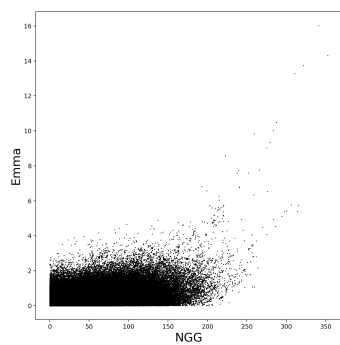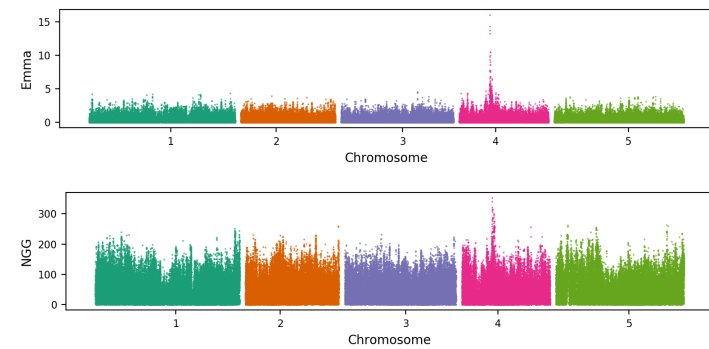

P31

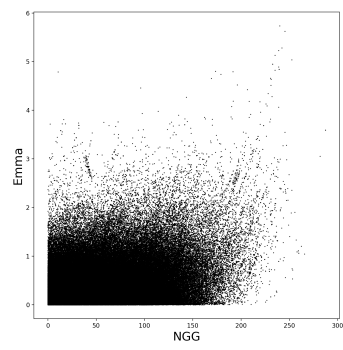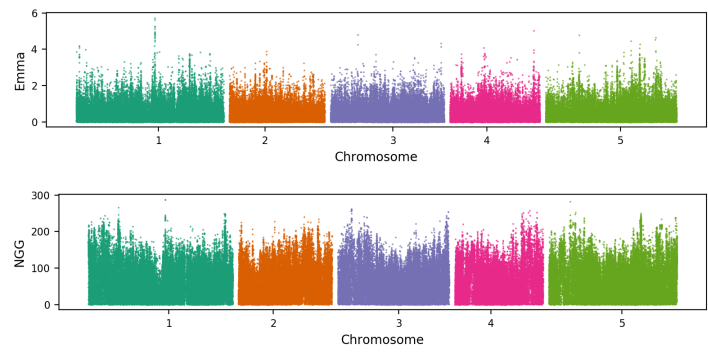

Rb85

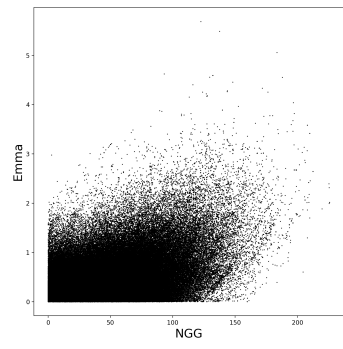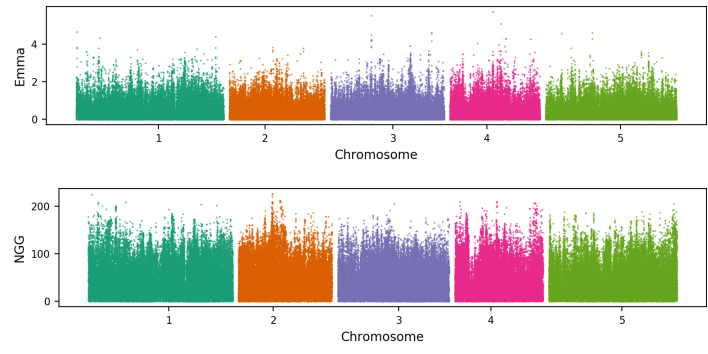

S34

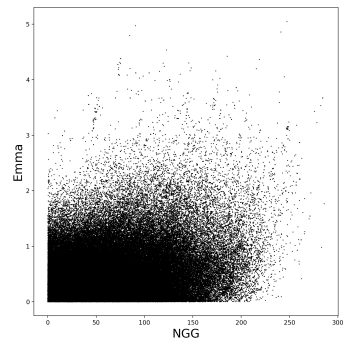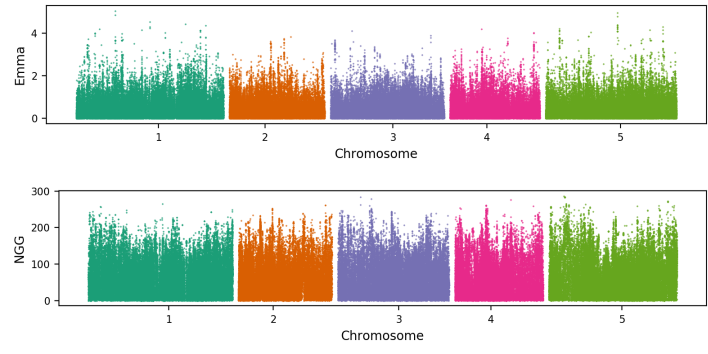

Se82

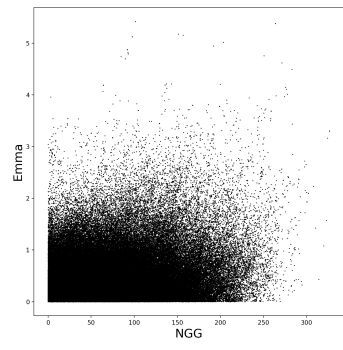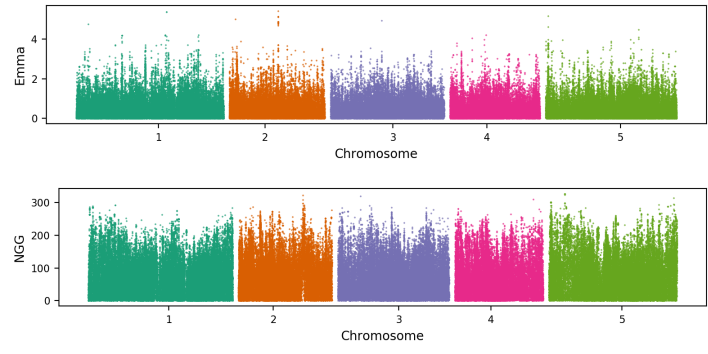

Sr88

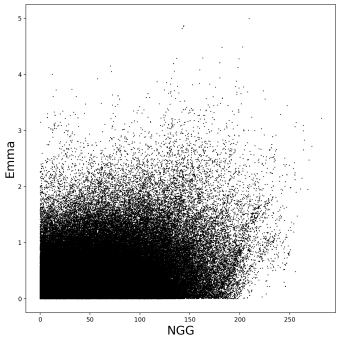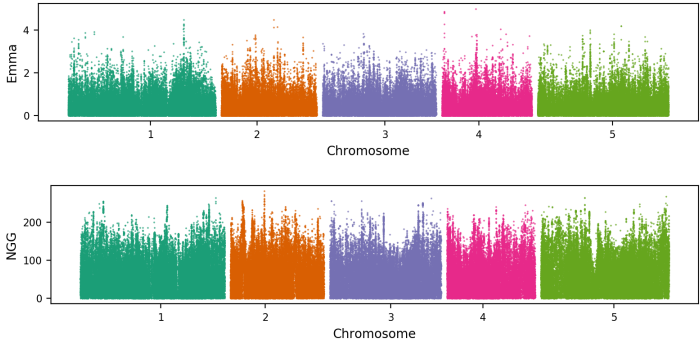

Zn66

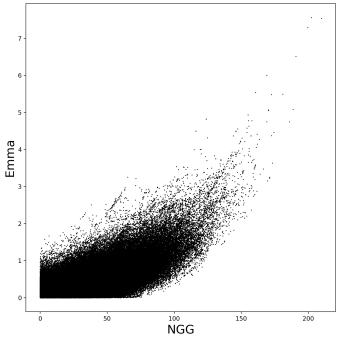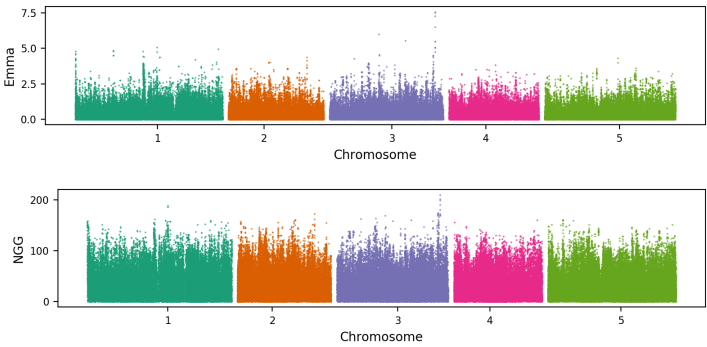

Supplement: Supplementary file 1 — Additional file 1. Additional text, figures and table. [file 13059_2024_3202_MOESM1_ESM.zip › Sup Figure 7.pdf]
