# Supplementary material for: Next-Gen GWAS: full 2D epistatic interaction maps retrieve part of missing heritability and improve phenotypic prediction
Source: Genome Biol. 2024 Mar 25;25:76. doi: 10.1186/s13059-024-03202-0 (PMC10962106; doi:10.1186/s13059-024-03202-0)
Supplement: Supplementary file 2 — Additional file 2. Review history. [file 13059_2024_3202_MOESM2_ESM.docx]

**1^st^ round**

**Reviewer 1**

Carre et al presents a method termed Next-Gen GWAS (NGG) that using GPU acceleration can perform GWAS like analyses (termed 1D). Using compressed sensing the method is up to 2 orders of magnitude faster than previous SOTA which allows estimation of epistatic interaction between SNVs across an entire genome (Arabidopsis thaliana). First the model is benchmarked on simulated data, identifying simulated epistatic effects. Hereafter, the model is applied to A. thaliana data performing trait association, then applied to identify epistatic effects and finally used for estimating heritability comparing 1D vs. 2D heritability.

Overall, the results are impressive - I was not aware of the compressed sensing techniques and it seems that these can be cleverly applied to estimate SNP-trait associations and SNP-SNP epigenetic effects. However, I found that the presentation of the method and results to be unstructured and confusing, and therefore I am not convinced of the claims.

1. The introduction is very short and does not provide credit towards previous work and state of the art. I.e. there needs to be more introduction on topics such as GWAS, missing heritability, the concept of epistasis, previous computational approaches for identifying epistasis and importantly to the techniques used in the paper such as compressed sensing. These are not, or only very briefly, introduced.

2. The "Results and Discussion" section seems to be a mix of introduction, methods, and results. Some topics are introduced in this section and not in the introduction, additionally methods are described in this section and not in the methods section. This makes it very hard to read and difficult to follow a thread in the manuscript. I think that modifying this will greatly improve the readability and understandability of the manuscript. Furthermore, consider shorter clearly defined paragraphs following a "question-results-take home message" flow.

3. The methods section is very short - this unacceptable brief. At minimum the simulated and genetic data sets must be described. How are the simulated data generated and processed. How are the real genetic datasets generated, how are they processed. Which traits are included, how was permGWAS run? Additionally, the text on heritability from the Results section could easily be moved here. These and probably several more paragraphs (also for ML part) are missing from the methods. Information is also missing on how to access the data and at the current state it is not possible at all to reproduce the analyses and results.

4. It is not clear to me exactly how this method (e.g., this new method called "model compression") works to achieve linear scaling w.r.t SNPs. While the prior text references techniques such as Compressed Sensing, it was unclear to me what the method is doing when I read the main text. Maybe it could be clarified in terms more amendable to biologists as this is submitted to Genome Biology.

5. Several of the figures are poor quality or are unreadable.
- Figure 2: missing values on axis or too small to read; Individual figures are not marked with letters making them harder to read.
- Figure 3: Unreadable axis legend; "Colored data points are emerging from the noise in a bootstrap procedure". What does this mean, what are the colors and how are they defined?
- Figure 4: Unreadable axes

6. The manuscript would be stronger with an overview figure, showing what data is being used (how many samples, how many in training/validation/test for machine learning methods), what is novel about the method (i.e., what is your proposed method actually doing that is interesting), the main result, etc. I found myself going back and forth in the manuscript to get a good overview.

7. Often claims are made without explicit stating the data/numbers supporting the claim. This is general across the manuscript and should be changed to include the actual evidence for the claims. For instance, pg 3 ln 47-56 has several claims but no actual numbers/results: "We found that NGG is quite resilient to noise but sensitive to the number of individuals used for the analysis (as discussed further, see remarks on Very High Dimension), as it radically improves for larger numbers of individuals (Supplementary Figure S1). We also measure NGG capacity to detect epistatic signals when the interacting SNPs are not randomly selected. Indeed, we simulated different scenario whereby a SNP can have a simple (1D) effect combined with interaction effects (2D). Again NGG is able to retrieve the simulated epistatic interactions even in more complex mixtures of simple and combinatorial effects (Sup Figure S2)."


Specific questions:

8. Do you have uncertainty estimates on your predictions? This was not clear to me.

9. I have never used permGWAS before, is it state-of-the-art for GWAS? What about SNPTESTv2? This method is, in our experience very fast.

10. What about other state-of-the-art GWAS testing, like REGENIE or GEMMA? Why are these not included? Comparing with these other methods that do interaction testing (such as regenie and interaction testing in PLINK/PLINK2) would also be of interest.

11. A "drastic procedure for variable selection" is mentioned, but not explained further.

12. When estimating how well the method scales w.r.t number of samples, why were the simulations stopped at 10K? In the context of modern human biobanks, it would be relevant to see if the method scales to, e.g., 1 million samples with 1 million+ markers. If it does scale, and the scaling is linear, is it correct to assume 1 million samples with 1 million SNPs should take around 600 seconds?

13. The documentation in the GitHub repository could be improved,, e.g., the list of main arguments under usage in ML_toolkit_NGG cuts in the middle.

14. If I understand correctly, one factor of the method is using GPUs for the GWAS—but I was unable to find that code implementation. Is that in the Heritability_estimation_NGG repository, or somewhere else? If yes, where does the GPU come into play there?

15. There are several peaks for flowering (Figure 4B) using NGG. Are they also found using traditional GWAS?

**Reviewer 2**

Carré et al present an approach for large-scale epistatic mapping in association studies. Their approach uses an application of compressed sensing to yield a sparse solution to the full 1D-2D interaction linear model. They focus on Arabidopsis GWAS and show that their approach yields similar 1D associations as standard methods while capturing some 2D effects that improve phenotype prediction.

This approach is clever and makes progress on an important problem in modern genetics. Ultimately I think that they demonstrate that their 2D maps capture phenotypic variation that is not captured by 1D mapping. However, I do have concerns about the methodology and evaluation that I would like to see addressed and I am also very concerned with the writing style and presentation. This is especially true given the broad readership of Genome Biology.

Scientific comments and concerns
- The definition of heritability (P2 L46, 49) is wrong. It should be H=var(f(X))/var(Y) and h=var(Xtheta)/var(Y).
- Figure 1 is difficult to understand. How do I differentiate between the true and estimated effects? How is the error shown? I see the purple is true but what is estimated for those signals? What is the 3D presentation adding that I couldn't get from a 2D heatmap? Relatedly -
- The simulations around Figure 1 never mention the sample size. And the number of SNPs is only given in the figure legend. In Figure S1, it seems like the estimation error is fairly large for moderate heritability SNPs when N=1000, which is roughly the number of samples in real data. And this is for only 100 SNPs. How is the error affected by having larger numbers of SNPs?
- Actual numbers in terms of estimation error, probability of retrieving simulated signals (power/FDR) are never given for any of these simulations. This makes it extremely difficult to evaluate the accuracy of the method.
- The authors claim that "major signals retrieved with EMMA algorithm are also retrieved by NGG" (p4 l26) needs stronger justification. This seems to be Figure S5 (? it is never mentioned). Some seem to have a tight relationship while others do not. I'd like to see some sort of overlap statistics across all of these phenotypes. What proportion of GWAS-significant signals from the EMMA analysis are detected by NGG? What may be driving anything missed or phenotypes where agreement is poor?
- The claim "It is noteworthy that the observed good correlation between EMMA and NGG results is also a guarantee that NGG performs genome population structure correction being at least comparable to mixed models" is extremely strong and not justified by the evidence presented. If this is true, it is very interesting, but I would need to see an entire set of simulations of data from mixed models to justify this claim. The mathematical justification given in that paragraph is plausible but not sufficient.
- In Figure 3 and the discussion p5 l38 - what is "support for the model"? Is this something produced in the estimation procedure? If so, it should be defined as part of an expanded section on the method (see below).
- In the discussion about VHD data, the authors note the sparsity should be around 50 (p5 l17). What happens when the true sparsity is larger or smaller than this? What do you miss? Does FDR increase if the true sparsity is less than specified? I'd like to see a large simulation addressing what happens here.
- Regarding the "missing heritability" retrieved (Figure 5) - the variance explained by this PCR approach is absolutely not the narrow sense heritability h2 and the difference between the variance explained by the model including epistatic interactions and not is absolutely not the missing heritability recovered. This is an interesting experiment, and it does demonstrate that there is phenotypic variability captured by the epistatic interactions that is not captured by the marginal interactions. However, the authors need to be more careful about the language here. There should be more discussion of what this quantity is and how it is related to the heritability. This is something that could go here or in a discussion section.

Presentation concerns
- The introduction is very short, and focuses primarily on the problem of missing heritability in humans, while the paper later focuses exclusively on Arabidopsis. It is possible that the role of epistasis is different in these species. Is there anything already known about epistasis in Arabidopsis or other model organisms specifically?
- The characterization of missing heritability in the abstract and introduction is slightly incorrect and makes too strong a claim about the role of epistasis. Specifically:
- "This mystery lies in the fact that a large proportion of phenotypes are not explained by unique or simple genomic modifications" - this doesn't actually make sense. A large proportion of phenotypes are not genetic - you probably mean heritability. Broad sense or narrow sense? Missing heritability can be characterized by both, and in the latter case we're seeing with some phenotypes this may literally just be a matter of sample size.
- "One has to invoke genetic interactions among different loci, also known as epistasis, to partly account for it" and "The missing heritability can at least partially be explained by variant interactions, ie: epistasis" this is likely to be true, at least for broad sense heritability, but this is also hypothesis that needs a citation where it is shown to be true or the language should be toned down to "likely to be".
- There are a large number of parenthetical remarks. These should be used sparingly if at all in this type of formal writing.
- In general, a lot of the language is informal and/or flowery, for example referring to the approach as "radically different". A particularly egregious example is the remark - "Having at hand a view of the full epistatic maps can be seen as a new route towards at least two kinds of developments. The first one is obviously experimental validations. These are very labor intensive and may require a very long time to precisely dissect epistatic interactions. These are under investigation but we wished to release our results in the light of the second." This just comes across as odd and would be better placed in a discussion section phrased very differently.
- There are a huge number of typographical errors
- I'd like to see a little more about the method in the section "The model and it's fast resolution". Specifically, a few sentences about the compressed sensing approach and the variable selection procedure would be helpful.
- The paper contains no discussion section aside from a few sentences at the end without it's own section. There should be a discussion section with at least
- Limitations of this approach/study
- Interpretation of results as compared to standard GWAS methods
- Opportunities for future work in this area

Other minor concerns
- The three types of simulated epistatic interactions (p4 l11) are never defined. These should at least be in the supplement.
- Figures s4, s5, and s6 are never mentioned in the main text.
- The claim "for certain phenotypes, NGG and EMMA converge towards a 2 relationship (Supplemental Figure S3)" is not Figure S3. Maybe this is S5?
- Maybe I'm missing it - but I don't actually see a github link for the NGG program itself? I see links for generating simulated data (GFIM) and doing the ML (ML_toolkit_NGG) and the heritability estimation (heritability_estimation_NGG). I also dont see any link to the visualization tool described.

**Reviewer 3**

This is a very interesting paper to develop a tool for epistatic interaction and application to Arabidopsis data. Investigation of epistatic interaction is very important but also challenging due to the large number of possible interactions. The authors seem to have developed a practical tool using the Compressive sensing framework and GPU power. I have the following relatively minor comments.

1. In the main manuscript, nearly nothing about the NGG algorithm is described. Only a very basic fact (two-way interaction is included in the model) is introduced. All the explanations are in the supplementary materials. Since the algorithm itself is very important, I suggest the authors provide a high-level summary of the algorithm in the main manuscript.

2. I expected the algorithm to produce a sparse estimate of \theta, but it doesn't look like this is true (Figure 2, Manhattan plot). Is it because of the piecewise-solving and averaging? The authors should clarify it.

3. The algorithm provides effect sizes only, not p-values, so the next question would be how to select a cut-point for the association. There should be a discussion about the FWER or FDR controls.

4. Frankly, the discussion on VHD and k (page6) is difficult to follow. My understanding is that in VHD, to estimate \theta well, the true \theta should be very sparse, like k<50 non-zero elements. Then the authors said in line 18 that they focused on 30 significant interactions. Is there any guarantee that although true \theta is not very sparse (like k > 30 ~50), the top 30 SNP effects are well estimated? It sounds like they are different problems.


Minor comments: Typos and Missing information make the paper difficult to read.

Page 2, Line 46: Heritability is var(F(x))/var(Y), not var(F(x))/var(\epsilon)

Page 4 Line 34: Supplementary Figure 5? Not 3?

Figure 1: Is the number of non-sparse element 5? Then it should be clearly written. If not, it also should be written that how many were missed.

Figure 2 and Supplementary Figure 5: I guess EMMA values in Manhattan and scatter plot is -log10 p-values? In Figure 2, Y-axis of Manhattan plot is shown, but other plots are not.
Figure 3:
"Colored data points are emerging from the noise in a bootstrap procedure comparable to permGWAS procedure". Not sure what it means? There are different colors, and the author should describe what each color means.
Page 5, line 38: support for the model x |SNP effect|: unclear.

**Authors’ response**

**Reviewer #1:** Carre et al presents a method termed Next-Gen GWAS (NGG) that using GPU acceleration can perform GWAS like analyses (termed 1D). Using compressed sensing the method is up to 2 orders of magnitude faster than previous SOTA which allows estimation of epistatic interaction between SNVs across an entire genome (Arabidopsis thaliana). First the model is benchmarked on simulated data, identifying simulated epistatic effects. Hereafter, the model is applied to A. thaliana data performing trait association, then applied to identify epistatic effects and finally used for estimating heritability comparing 1D vs. 2D heritability.

Overall, the results are impressive - I was not aware of the compressed sensing techniques and it seems that these can be cleverly applied to estimate SNP-trait associations and SNP-SNP epigenetic effects. However, I found that the presentation of the method and results to be unstructured and confusing, and therefore I am not convinced of the claims.

Would like to thank the reviewers for the compliment and we hope that the revised version will convince the reviewers that our claims are sound.

1. The introduction is very short and does not provide credit towards previous work and state of the art. I.e. there needs to be more introduction on topics such as GWAS, missing heritability, the concept of epistasis, previous computational approaches for identifying epistasis and importantly to the techniques used in the paper such as compressed sensing. These are not, or only very briefly, introduced.

We understood the critique and agreed with it. We now provide an extended version of the introduction.

2. The "Results and Discussion" section seems to be a mix of introduction, methods, and results. Some topics are introduced in this section and not in the introduction, additionally methods are described in this section and not in the methods section. This makes it very hard to read and difficult to follow a thread in the manuscript. I think that modifying this will greatly improve the readability and understandability of the manuscript. Furthermore, consider shorter clearly defined paragraphs following a "question-results-take home message" flow.

Since the Method is intrinsically the point of this work we wanted to present more the thread of ideas that lead to the building of NGG. That’s why the method section is short because we believe in some ways that the whole paper is a method paper. But we understand that this writing may not be appreciated. We thus did our best to improve the introduction early on to

provide key concepts and we also tried to make the rest of the manuscript more easy to read by providing introductory sentences and conclusions for each paragraph.

3. The methods section is very short - this unacceptable brief. At minimum the simulated and genetic data sets must be described. How are the simulated data generated and processed. How are the real genetic datasets generated, how are they processed. Which traits are included, how was permGWAS run? Additionally, the text on heritability from the Results section could easily be moved here. These and probably several more paragraphs (also for ML part) are missing from the methods. Information is also missing on how to access the data and at the current state it is not possible at all to reproduce the analyses and results.

Here again we understand the critique on the form and we did our best to provide a maximum of information in the method section and throughout the whole manuscript. On the critique concerning the reproducibility we clearly less understand since we provided the code and full description of the parallelization method in the sup text info. This work can be totally reproduced with the provided information. However, in order to comply with the reviewer's remark we tried to expand the method section and also now provide code for the GPU parallelized version (in *Python*).

4. It is not clear to me exactly how this method (e.g., this new method called "model compression") works to achieve linear scaling w.r.t SNPs. While the prior text references techniques such as Compressed Sensing, it was unclear to me what the method is doing when I read the main text. Maybe it could be clarified in terms more amenable to biologists as this is submitted to Genome Biology.

This has been raised as well by another reviewer and we agree with the critique. We now provide a new Figure (Fig. 1) as well as a full new paragraph meant to clarify this point. We would also like to point the reviewer to the extended description of the mathematical method provided originally as a supplemental text.

5. Several of the figures are poor quality or are unreadable.

- Figure 2: missing values on axis or too small to read; Individual figures are not marked with letters making them harder to read.

- Figure 3: Unreadable axis legend; "Colored data points are emerging from the noise in a bootstrap procedure". What does this mean, what are the colors and how are they defined? - Figure 4: Unreadable axes

We thank the reviewer for pointing this out. We did correct the figures and provide more details. We also believe that the compression algorithm that makes the PDF is part of the discrepancy.

6. The manuscript would be stronger with an overview figure, showing what data is being used (how many samples, how many in training/validation/test for machine learning methods), what is novel about the method (i.e., what is your proposed method actually doing that is interesting),

the main result, etc. I found myself going back and forth in the manuscript to get a good overview.

As suggested, a new figure (Fig. 1) has been built to answer this particular request and the one before (question 4). We also consolidated the figure legends to make clearer explanations. We hope that this will meet reviewer #1 expectations.

7. Often claims are made without explicit stating the data/numbers supporting the claim. This is general across the manuscript and should be changed to include the actual evidence for the claims. For instance,

pg 3 ln 47-56 has several claims but no actual numbers/results: "We found that NGG is quite resilient to noise but sensitive to the number of individuals used for the analysis (as discussed further, see remarks on Very High Dimension), as it radically improves for larger numbers of individuals (Supplementary Figure S1).

We also measure NGG capacity to detect epistatic signals when the interacting SNPs are not randomly selected. Indeed, we simulated different scenario whereby a SNP can have a simple (1D) effect combined with interaction effects (2D). Again NGG is able to retrieve the simulated epistatic interactions even in more complex mixtures of simple and combinatorial effects (Sup Figure S2)."

We understand this remark. We have now rephrased to add numbers. We also now provide new results concerning these claims (New Figures 2E, 2F) as we provide the recall values for 0.5M epistatic interactions for increasing values of h^2^ and increasing number of individuals.

Specific questions:

8. Do you have uncertainty estimates on your predictions? This was not clear to me.

New Figures 2E, 2F provides estimation for this. In a particular context of 350k^2 interactions and expected heritability measured Figure 6B, we estimate that 50% of the top 10 score is meant to be a real signal.

9. I have never used permGWAS before, is it state-of-the-art for GWAS? What about SNPTESTv2? This method is, in our experience very fast.

We have compared SNPTESTv2 and permGWAS. The latter is faster. Our analysis thus holds as we compare the faster algorithms to date.

Please refer to the response to point 10 below for more details.

10. What about other state-of-the-art GWAS testing, like REGENIE or GEMMA? Why are these not included? Comparing with these other methods that do interaction testing (such as regenie and interaction testing in PLINK/PLINK2) would also be of interest.

To address the comments 9 and 10, we conducted tests on an identical dataset (1 million SNPs, 1000 individuals) using GEMMA, SNPTESTv2, and permGWAS. Their respective runtimes were 29, 23, and 6 minutes, as now documented on page 6 of the manuscript. Given that NGG operates approximately 100 times faster than permGWAS, which is itself about 4 times quicker than earlier methods, we believe a comprehensive comparison may not be necessary. Such an analysis would likely be time-consuming and expensive to perform.

11. A "drastic procedure for variable selection" is mentioned, but not explained further.

An explanation of the variable selection procedure was added and is copied below ^

“The variable selection procedure is made of two steps : (i) the effects collected in θ are ranked in decreasing order and (ii) only the N* first largest effects are retrieved. Here the choice of N* follows the lines of [22] (see e.g. proposition 6.2 : our N* stands for the k in the cited article) since we are in a “very high dimensional“ framework in the sense of data scientists and statisticians.”

12. When estimating how well the method scales w.r.t number of samples, why were the simulations stopped at 10K? In the context of modern human biobanks, it would be relevant to see if the method scales to, e.g., 1 million samples with 1 million+ markers. If it does scale, and the scaling is linear, is it correct to assume 1 million samples with 1 million SNPs should take around 600 seconds?

Yes it is correct. We understand the remark and we would like to emphasize that we did run NGG on real datasets being 350k^2/2 > 60.10^9^interactions (Figure 6) in ~45 min. However analyzing epistatic maps and producing simulated data of such a large dataset is a different task. We would like to remind the reviewer that to build the epistatic signal in the simulation process we actually need to build the Z matrix. As when we solve it (via NGG) we don’t have to fully build it but instead we make the interactions on the fly (see Sup Text).

Despite this difficulty and to further convince Reviewer #2, we ran a new set of simulation and experiments on 1000 x 1000 SNP interactions (=500K) and with an increasing number of individuals (25 t0 10k) (New Figure 2E and 2F). We also analyze the recall we obtain on the 10 and 100 top signals reported by NGG. We demonstrate that the top signals contain the simulated epistatic signals.

In other words the whole work on NGG is meant to optimize speed in signal retrieval (theta) and not in simulation algorithms. We hope that this clarifies this point.

13. The documentation in the GitHub repository could be improved,, e.g., the list of main arguments under usage in ML_toolkit_NGG cuts in the middle.

We have modified this documentation and now provide a new code.

14. If I understand correctly, one factor of the method is using GPUs for the GWAS—but I was unable to find that code implementation. Is that in the Heritability_estimation_NGG repository, or somewhere else? If yes, where does the GPU come into play there?

In the first version of the work we provided the full scheme of the NGG algorithm and its implementation strategy in Sup Text information (algorithmic details).

To comply with reviewer #2 remark we now provide a Python GPU parallelized code to help to understand our strategy to parallelize the code of GPU architecture.

15. There are several peaks for flowering (Figure 4B) using NGG. Are they also found using traditional GWAS?

For this particular phenotype (flowering) more than 50% of the epistatic peaks do not present a signal in the diagonal (traditional) GWAS. But this is not a general rule as we can observe that Phosphorus epistatic signal is a “pure” epistatic effect as, when not combined the SNPs don’t have an effect by themselves (Figure 5B). This is a very good illustration that brute force approach as NGG can pinpoint totally new epistatic markers that may have been missed by traditional approaches that use variable selection to reduce problem size.

**Reviewer #2:** Carré et al present an approach for large-scale epistatic mapping in association studies. Their approach uses an application of compressed sensing to yield a sparse solution to the full 1D-2D interaction linear model. They focus on Arabidopsis GWAS and show that their approach yields similar 1D associations as standard methods while capturing some 2D effects that improve phenotype prediction.

This approach is clever and makes progress on an important problem in modern genetics. Ultimately I think that they demonstrate that their 2D maps capture phenotypic variation that is not captured by 1D mapping. However, I do have concerns about the methodology and evaluation that I would like to see addressed and I am also very concerned with the writing style and presentation. This is especially true given the broad readership of Genome Biology.

Scientific comments and concerns

- The definition of heritability (P2 L46, 49) is wrong. It should be H=var(f(X))/var(Y) and h=var(Xtheta)/var(Y).

We thank the reviewer for pointing out this obvious mistake. This was a typo as may be

deduced from the sentence following the definition of ℎ. “At last when the estimation method is Ordinary Least Squares (OLS) or some of its (regularized/penalized) variants, the definition above matches the classical R2 and adjusted R2.”. We double checked the code provided in the git repository and we confirmed that this was indeed a typo with no consequences on the computational results. We thank the reviewer for pointing this out.

- Figure 1 is difficult to understand. How do I differentiate between the true and estimated effects? How is the error shown? I see the purple is true but what is estimated for those signals? What is the 3D presentation adding that I couldn't get from a 2D heatmap? Relatedly -

We understand this could be confusing. We believe that 3D presentation helps to visualize at the same time the position of the simulated signals (purple) and the predicted effect of each of the SNPs and SNP interactions. This may help capturing by eye the recall and the noise in the landscape of interactions. We also now provide new figures (2E, EF) measuring the progression of the recall for a large number of SNP interactions and an increasing number of individuals and an increasing h2. We hope this will clarify the situation.

- The simulations around Figure 1 never mention the sample size. And the number of SNPs is only given in the figure legend. In Figure S1, it seems like the estimation error is fairly large for moderate heritability SNPs when N=1000, which is roughly the number of samples in real data. And this is for only 100 SNPs. How is the error affected by having larger numbers of SNPs?

We agree that this was missing information and it is now reported in the figure legend. We also provide more simulations and results are reported in Figure 2E and 2F.

- Actual numbers in terms of estimation error, probability of retrieving simulated signals (power/FDR) are never given for any of these simulations. This makes it extremely difficult to evaluate the accuracy of the method.

We agreed on this. As mentioned above we also now provide Figure 2E and 2F evaluating recall for large number of SNPs and estimate our capacity of signal retrieval.

- The authors claim that "major signals retrieved with EMMA algorithm are also retrieved by NGG" (p4 l26) needs stronger justification. This seems to be Figure S5 (? it is never mentioned). Some seem to have a tight relationship while others do not. I'd like to see some sort of overlap statistics across all of these phenotypes. What proportion of GWAS-significant signals from the EMMA analysis are detected by NGG? What may be driving anything missed or phenotypes where agreement is poor?

We now provide a new figure to present this overlap for every single phenotype as proposed by reviewer #2. This can be found in new Sup. Figure 5. We now discuss the congruence between both methods in the new discussion section.

- The claim "It is noteworthy that the observed good correlation between EMMA and NGG results is also a guarantee that NGG performs genome population structure correction being at least comparable to mixed models" is extremely strong and not justified by the evidence presented. If this is true, it is very interesting, but I would need to see an entire set of simulations of data from mixed models to justify this claim. The mathematical justification given in that paragraph is plausible but not sufficient.

Understand this remark. We did tone down our claims to comply with the reviewer remarks.

- In Figure 3 and the discussion p5 l38 - what is "support for the model"? Is this something produced in the estimation procedure? If so, it should be defined as part of an expanded section on the method (see below).

We apologize if this was not clear. Support of the model is just a vector of 0 and 1 ignoring the strength of the signal but rather is a signal is there or not. We have clarified this in the legend.

- In the discussion about VHD data, the authors note the sparsity should be around 50 (p5 l17). What happens when the true sparsity is larger or smaller than this? What do you miss? Does FDR increase if the true sparsity is less than specified? I'd like to see a large simulation addressing what happens here.

We discuss this point now in the new discussion section. Although we did perform new simulations for a high number of SNP interactions (Figure 2E,F) we believe that testing different levels of sparsity falls outside of the scope of this work. Indeed, as discussed in the new Discussion section we think that the fact that NGG performs block estimation of effects and repeat this several times (see Sup text 1) this problem tends to be mitigated. As thus we think that we have demonstrated that NGG is able to retrieve true epistatic signals we would rather leave this study for further investigation.

- Regarding the "missing heritability" retrieved (Figure 5) - the variance explained by this PCR approach is absolutely not the narrow sense heritability h2 and the difference between the variance explained by the model including epistatic interactions and not is absolutely not the missing heritability recovered. This is an interesting experiment, and it does demonstrate that there is phenotypic variability captured by the epistatic interactions that is not captured by the marginal interactions. However, the authors need to be more careful about the language here. There should be more discussion of what this quantity is and how it is related to the heritability. This is something that could go here or in a discussion section.

The referee is right : we did not strictly plot here the narrow sense heritability and we may not have been careful enough with our language. We are rather willing to show the added value of our approach in order to estimate -imperfectly yet- the unknown true value of h2.

Discussion :

Narrow sense heritability depends on the definition of the linear model investigated. Taking our Model 1 for instance, the slope parameter may be subject to mathematical constraints (boundedness, sparsity, bayesian prior…) that will influence the computation and the value of h2. Here the only assumption is sparsity. However estimating correctly h2 is a difficult and delicate question. The theoretical definition of h2 matches the definition of the classical R2 and it is well known that R2 is biased and tends to inflate when the number of predictors is large. A solution to estimate an unbiased h2 may be to balance the bias by computing the adjusted R2. Unfortunately the adjusted R2 is not defined when the number of predictors is larger than the sample size, like here. A new definition could be to modify the adjusted R2 and to replace the number of predictors p by the sparsity k (mathematically all this comes down to change the penalty applied to the R2) :

��^2^_������_ = 1 − (1 − ��^2^)(^��−1^

_��−��−1_~~)~~

But once again we face a problem : the sparsity index k is unknown.

Our method tries to overcome these issues : PCA-regression allows us to compute an approximation of the numerator in the definition of h2 (at least as good as PCA may capture the variance of X). But this time the value of narrow sense missing heritability is underestimated instead of being overestimated. The nonlinear machinery of PCA regression deviates somehow from the initial definition of h2 but may be easily interpreted and provides realistic numerical evaluations.

Presentation concerns

- The introduction is very short, and focuses primarily on the problem of missing heritability in humans, while the paper later focuses exclusively on Arabidopsis. It is possible that the role of epistasis is different in these species. Is there anything already known about epistasis in Arabidopsis or other model organisms specifically?

We agree with this remark and it has also be raised by reviewer #1. We now provide a deeply modified version of the introduction trying to acknowledge previous attempts to measure

- The characterization of missing heritability in the abstract and introduction is slightly incorrect and makes too strong a claim about the role of epistasis. Specifically: "This mystery lies in the fact that a large proportion of phenotypes are not explained by unique or simple genomic modifications" - this doesn't actually make sense. A large proportion of phenotypes are not genetic - you probably mean heritability. Broad sense or narrow sense? Missing heritability can be characterized by both, and in the latter case we're seeing with some phenotypes this may literally just be a matter of sample size.

We agree with this statement. We rephrase accordingly in order to be very precise. Also we would like to point now that we added a discussion about this. We bring the attention of the reader to the fact that some phenotypes (Figure 5b) don’t benefit from epistatic signal in the predictability of the model.

- "One has to invoke genetic interactions among different loci, also known as epistasis, to partly account for it" and "The missing heritability can at least partially be explained by variant interactions, ie: epistasis" this is likely to be true, at least for broad sense heritability, but this is also hypothesis that needs a citation where it is shown to be true or the language should be toned down to "likely to be".

We agree with this precision and the text has been corrected according to reviewer remark.

- There are a large number of parenthetical remarks. These should be used sparingly if at all in this type of formal writing.

We did our best to reduce these. It is part of our style of writing. Maybe the editorial team will get rid of these at the proof stage (if any).

- In general, a lot of the language is informal and/or flowery, for example referring to the approach as "radically different". A particularly egregious example is the remark - "Having at hand a view of the full epistatic maps can be seen as a new route towards at least two kinds of developments. The first one is obviously experimental validations. These are very labor intensive and may require a very long time to precisely dissect epistatic interactions. These are under investigation but we wished to release our results in the light of the second." This just comes across as odd and would be better placed in a discussion section phrased very differently.

This has been rephrased into more formal english.

- There are a huge number of typographical errors

We did our best to mitigate this in the revised document.

- I'd like to see a little more about the method in the section "The model and it's fast resolution". Specifically, a few sentences about the compressed sensing approach and the variable selection procedure would be helpful.

This has been implemented in the introduction. We hope this will meet the reviewer's expectations.

- The paper contains no discussion section aside from a few sentences at the end without it's own section. There should be a discussion section with at least

- Limitations of this approach/study

- Interpretation of results as compared to standard GWAS methods

- Opportunities for future work in this area

A discussion part has been made following reviewer 1 and 2 instructions.

Other minor concerns

- The three types of simulated epistatic interactions (p4 l11) are never defined. These should at least be in the supplement.

We now define this and also cite the reference fully describing these interactions. The code for simulation is also provided.

- Figures s4, s5, and s6 are never mentioned in the main text.

Thanks for pointing this out. We did correct this.

- The claim "for certain phenotypes, NGG and EMMA converge towards a 2 relationship (Supplemental Figure S3)" is not Figure S3. Maybe this is S5?

Yes indeed, thank you.

- Maybe I'm missing it - but I don't actually see a github link for the

? I see links for generating simulated data (GFIM) and doing the ML (ML_toolkit_NGG) and the heritability estimation (heritability_estimation_NGG). I also don't see any link to the visualization tool described.

We now provide a link to download the visualization tool **{here}**.

http://luciol.reviewing.process.bionomeex.tech/

**Reviewer #3:** This is a very interesting paper to develop a tool for epistatic interaction and application to Arabidopsis data. Investigation of epistatic interaction is very important but also challenging due to the large number of possible interactions. The authors seem to have developed a practical tool using the Compressive sensing framework and GPU power. I have the following relatively minor comments.

We thank the reviewer for such encouraging comments.

1. In the main manuscript, nearly nothing about the NGG algorithm is described. Only a very basic fact (two-way interaction is included in the model) is introduced. All the explanations are in the supplementary materials. Since the algorithm itself is very important, I suggest the authors provide a high-level summary of the algorithm in the main manuscript.

We agree with this remark. We now provide a new figure as this claim is also common with all other reviewers.

2. I expected the algorithm to produce a sparse estimate of \theta, but it doesn't look like this is true (Figure 2, Manhattan plot). Is it because of the piecewise-solving and averaging? The authors should clarify it.

Yes exactly. It is sparse for each pack of SNP being evaluated. But since this procedure is repeated on several packages which dimension is defined by the GPU capacity, then it converges towards an averaged non purely sparse results. This is now mentioned on the text.

3. The algorithm provides effect sizes only, not p-values, so the next question would be how to select a cut-point for the association. There should be a discussion about the FWER or FDR controls.

We agree and now discuss this point following the reviewer's will. This has been added to the new discussion section.

Bootstrap

4. Frankly, the discussion on VHD and k (page6) is difficult to follow. My understanding is that in VHD, to estimate \theta well, the true \theta should be very sparse, like k<50 non-zero elements. Then the authors said in line 18 that they focused on 30 significant interactions. Is there any

guarantee that although true \theta is not very sparse (like k > 30 ~50), the top 30 SNP effects are well estimated? It sounds like they are different problems.

We understood the critique and we tried to make our point clearer. In particular now this is discussed in the new discussion section. In short, yes the reviewer is right. For 60B interactions we estimate that 30-50 interactions can be retrieved. However, it is slightly more complicated since the algorithm is built to solve the problem by blocks that fit in GPUs. And it does this multiple times (see Sup Text). As such this rule applies to each block. Then by averaging the signal we can expect to overpass this limitation. This point is now discussed in the new discussion section and is not the central point of this work and will surely need further investigations.

Minor comments: Typos and Missing information make the paper difficult to read. Several corrections pass have been performed to mitigate this.

Page 2, Line 46: Heritability is var(F(x))/var(Y), not var(F(x))/var(\epsilon) We fixed this thank you for pointing this out.

Page 4 Line 34: Supplementary Figure 5? Not 3?

We fixed and renumbered many figures due the new figure 1 and new Sup figure 5.

Figure 1: Is the number of non-sparse element 5? Then it should be clearly written. If not, it also should be written that how many were missed.

Yes the number of elements to retrieve is 5. We now write this in the legend.

Figure 2 and Supplementary Figure 5: I guess EMMA values in Manhattan and scatter plot is -log10 p-values? In Figure 2, Y-axis of Manhattan plot is shown, but other plots are not. Figure 3:

"Colored data points are emerging from the noise in a bootstrap procedure comparable to permGWAS procedure". Not sure what it means? There are different colors, and the author should describe what each color means.

Page 5, line 38: support for the model x |SNP effect|: unclear.

We clarified these points. We thank the reviewer for the careful reading of this work.

**2nd round**

**Reviewer 1**

The authors did a good job in meeting the critique, in particular adding Figure 1 has improved the intuitive understanding of the approach.

**Reviewer 2**

I thank the authors for their thorough response and revision. The new manuscript is much improved. However I do still have some concerns about the presentation of the claims in the manuscript:

- The addition of Figure 2E and F does help clarify the accuracy of the method. However something seems off with 2E. If there are 10 true effects, and this is Recall within the top 10 effects, shouldn’t the recall always be integer/10? Yet values in the table all have 2 significant digits.
I still think the discussion around handling population structure is too strong. I suggest something like the following: “The observed good correlation between EMMA and NGG results may indicate that NGG performs genome population structure correction comparably to mixed models. Mathematically, population structure correction is explained… . However, further simulations incorporating diverse genetic architectures and models of population structure would be required to fully validate this observation.”

- I still think the discussion around missing heritability confuses the concept. I don’t think that simply putting “missing heritability” in quotes makes it clear that this quantity is not the same as standard heritability definitions. This is further confused by the authors continuing to use h2 in their presentation here. As the authors point out in their response to reviewers, and as was my point in this critique, regression R2 and the actual h2 are very different in practice, which is why mixed model variance components or linkage scores are usually used to calculate it. I suggest text similar to the following: “We set out to understand the “missing heritability” explained by our recovered epistatic interactions. To do so, we calculated the increased variance explained [etc, regarding the PCR]. We note that this is not strictly the missing heritability recovered, [discussion of the relationship between these quantities].”

- Regarding the response to my comment on VHD data - it seems the authors are implying that a block and restitch scheme is needed to analyze genome-wide data, and that this is what is driving the limitation in terms of # of SNPs in simulation, which tops out at 1k rather than the O(100k) in real data. This seems to be just due to the limitation of fitting the data matrix in GPU memory. This makes sense, and I guess is now explained to some extent in the “Limitations” section of the discussion. However, given the general language in the text around solving the pairwise testing problem it should be clear much earlier that in genome-wide data the genome needs to be grouped into ~1k? (whats the actual maximum?) SNP blocks that are run sequentially. Given this, you aren’t actually getting genome-wide epistatic maps. That’s perfectly fine and this still represents an advancement, but this limitation needs to be made very clear very early in the manuscript.

Some minor comments:
P2 l56 - I assume they mean predominantly zero elements?
P3 l46 - given the readership of Genome Biology, I would explain the definition of the subset operator (or just state this in words without using the operator.)
The text in a number of the main figures is too small to read

**Reviewer 3**

The authors addressed my previous comments.
Some minor issues need to be addressed.

- line indent for the first line of a paragraph and line space between paragraphs are not consistent.
- Page 4, line 35, (1000x1000): This is described in the figure, but you also need to describe it in the text.
- Page 8, line 57 paragraph: Can it be moved to the discussion?
- Figure 1, Why the computation complexity of EMMA is p^3? Did you simply consider calculating the inverse of a pxp matrix? Is EMMA required to do it? I guess EMMA was done marker by marker, so do not need to convert a pxp matrix. You need to report the actual computation complexity of EMMA.

**Authors’ response**

Reviewer #1: The authors did a good job in meeting the critique, in particular adding Figure 1 has improved the intuitive understanding of the approach.

We thank the reviewer for this positive comment.

Reviewer #2: I thank the authors for their thorough response and revision. The new manuscript is much improved. However I do still have some concerns about the presentation of the claims in the manuscript:

- The addition of Figure 2E and F does help clarify the accuracy of the method. However something seems off with 2E. If there are 10 true effects, and this is Recall within the top 10 effects, shouldn't the recall always be integer/10? Yet values in the table all have 2 significant digits.

I still think the discussion around handling population structure is too strong. I suggest something like the following: "The observed good correlation between EMMA and NGG results may indicate that NGG performs genome population structure correction comparably to mixed models. Mathematically, population structure correction is explained… . However, further simulations incorporating diverse genetic architectures and models of population structure would be required to fully validate this observation."

We incorporated these suggestions to tone down the message indeed.

- I still think the discussion around missing heritability confuses the concept. I don't think that simply putting "missing heritability" in quotes makes it clear that this quantity is not the same as standard heritability definitions. This is further confused by the authors continuing to use h2 in their presentation here. As the authors point out in their response to reviewers, and as was my point in this critique, regression R2 and the actual h2 are very different in practice, which is why mixed model variance components or linkage scores are usually used to calculate it. I suggest text similar to the following: "We set out to understand the "missing heritability" explained by our recovered epistatic interactions. To do so, we calculated the increased variance explained [etc, regarding the PCR]. We note that this is not strictly the missing heritability recovered, [discussion of the relationship between these quantities]."

We incorporated these suggestions to make the distinction between h2 and R2.

- Regarding the response to my comment on VHD data - it seems the authors are implying that a block and restitch scheme is needed to analyze genome-wide data, and that this is what is driving the limitation in terms of # of SNPs in simulation, which tops out at 1k rather than the O(100k) in real data. This seems to be just due to the limitation of fitting the data matrix in GPU memory. This makes sense, and I guess is now explained to some extent in the "Limitations" section of the discussion.

Indeed, it is fully described in the Sup Text as well and can be seen in the code provided.

However, given the general language in the text around solving the pairwise testing problem it should be clear much earlier that in genome-wide data the genome needs to be grouped into ~1k? (whats the actual maximum?) SNP blocks that are run sequentially.

Indeed, the size of the block is calculated to maximize GPU memory usage. This is described in Sup Tex1. Concerning “SNP blocks that are run sequentially.” actually they are run in GPUs thus in parallel.

Given this, you aren't actually getting genome-wide epistatic maps. That's perfectly fine and this still represents an advancement, but this limitation needs to be made very clear very early in the manuscript.

We are not sure what the reviewer means by this last sentence. Figure 5B shows that our approach can detect “pure” epistatic signals for which SNP alone does not influence the phenotype value and that it is only when combined with another SNP that the effect appears.

Some minor comments:

P2 l56 - I assume they mean predominantly zero elements?

P3 l46 - given the readership of Genome Biology, I would explain the definition of the subset operator (or just state this in words without using the operator.)

The text in a number of the main figures is too small to read

We fixed these points accordingly. Thank you for pointing these out.

Reviewer #3: The authors addressed my previous comments.

Some minor issues need to be addressed.

- line indent for the first line of a paragraph and line space between paragraphs are not consistent.

- Page 4, line 35, (1000x1000): This is described in the figure, but you also need to describe it in the text.

Thanks for mentioning this. We fixed this.

- Page 8, line 57 paragraph: Can it be moved to the discussion?

We understand the proposal but we apologize as we rather keep this idea here because we believe it motivated the last part of the result section concerning phenotype prediction.

- Figure 1, Why the computation complexity of EMMA is p^3? Did you simply consider calculating the inverse of a pxp matrix? Is EMMA required to do it? I guess EMMA was done marker by marker, so do not need to convert a pxp matrix. You need to report the actual computation complexity of EMMA.

Yes, this complexity takes into account the computation of the kinship matrix, which drives

the whole complexity for this number of SNP. We did change the wording in the manuscript

and figure legend to be more explicit
